# Detailed Characterization of Pore Results of Continental Shale Reservoir in Fengcheng Formation, Mahu Sag

**DOI:** 10.1021/acsomega.4c02056

**Published:** 2024-05-14

**Authors:** Zhengchen Zhang, Kouqi Liu, Zhenlin Wang, Feifei Luo, Hong Zhang

**Affiliations:** † Institute of Energy, School of Earth and Space Sciences, Peking University, Beijing 100084, China; ‡ Exploration and Development Research Institute of CNPC, Xinjiang Oilfield Company, Karamay 834000, China

## Abstract

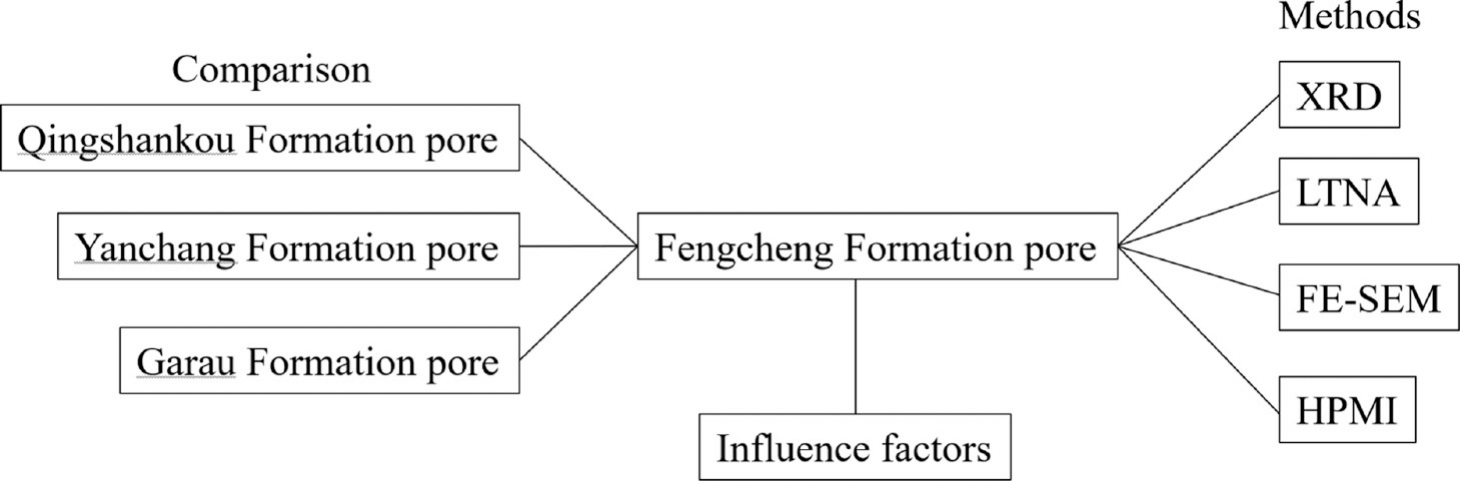

The exploration potential of shale oil in the Fengcheng Formation of the Mahu Sag, located in the Junggar Basin, is significant. However, there is a notable dearth of research on shale oil within this formation. This study addresses this gap by focusing on the pore structure and associated development factors of the Fengcheng Formation in Mahu Sag. A total of 113 samples from well X of the Fengcheng Formation were meticulously selected for analysis. The mineral components and pore structure of these samples were characterized using advanced techniques, including X-ray diffraction (XRD), low-temperature nitrogen adsorption (LTNA), high-pressure mercury injection (HPMI), and scanning electron microscopy (SEM). The findings reveal that, despite a relatively low content of clay minerals in the Fengcheng Formation, their presence is intricately linked to pore development. The high content of feldspar in the formation contributes significantly to the formation of clay minerals through dissolution processes. This dual influence plays a crucial role in shaping the overall pore development within the Fengcheng Formation. In addition, a comparative analysis was conducted with shale samples from other wells within the Fengcheng Formation in Mahu Sag, as well as from different basins, such as the Songliao Basin and Ordos Basin. Different from Qingshankou Formation in Songliao Basin and Yanchang Formation in Ordos Basin, the samples from Fengcheng Formation in Mahu Sag are composed of a large number of felic minerals and carbonate minerals with less clay minerals. This study underscores the paramount importance of mineral components and their respective content in influencing the pore development of shale oil reservoirs. The unique characteristics of the Fengcheng Formation in Mahu Sag, as revealed through comprehensive analyses, contribute valuable insights to the understanding of shale oil exploration potential in the Junggar Basin.

## 1 Introduction

Shale oil, as an important unconventional resource, has huge exploration potential. The shale oil and gas revolution in North America has attracted more and more attention.^1^ Unlike North America, shale oil in China mostly exists in lacustrine facies with complex geological conditions and uneven geochemical characteristics.^2,3^ The geological nature of continental shales in China, in comparison to North American marine shales, is marked by elevated pore structure complexity, pronounced heterogeneity, and limited organic pore development.^4−6^ To date, China has successfully achieved commercial development of continental shale oil in silt-carbonate interbeds within shale units, such as Ordos Basin Chang 7 Member and Songliao Basin Qingshankou Formation. The Fengcheng Formation, located in Mahu Sag, is considered to be the source area (rich in organic matter) and shale oil reservoir (rich in liquid) of Mahu Sag, and is currently the focus of shale oil research in this area.^7^ This academic exploration contributes to a deeper understanding of the unique characteristics and commercial potential of shale oil in the Mahu Sag area.

The pore structure of shale oil reservoirs plays a pivotal role as a carrier space and flow channel for hydrocarbons, significantly influencing the identification of sweet spots within shale reservoirs. At present, a large number of studies have reported that the pore structure of shale reservoir is affected by many factors, such as lithofacies, thermal maturity, mineral composition, organic matter (OM) content, OM composition, mineral transformation, and diagenesis.^8−11^ Based on Loucks’ classification of pores, shale pores can be divided into organic pores, intergranular pores, and intragranular pores. Notably, organic pores are the most important pore types in marine shales.,^9,12−17^ whereas continental shale is mainly composed of intergranular pores and intragranular pores.^17−22^ The Fengcheng Formation, recognized as the oldest high-quality alkaline lacustrine source rock, exhibits multistage efficient hydrocarbon generation, high yield, and significant shale oil potential.^23,24^ The large amount of alkaline minerals, the unique alkaline lacustrine sedimentary, and paleoenvironmental evolution history of Fengcheng Formation may affect the pore structure, and the interaction between oil and rock may further affect the oil enrichment and mobility.^25^ The formation has multistage efficient hydrocarbon generation, high hydrocarbon yield, and significant shale oil potential. Compared with other continental lacustrine shale, this shale holds heightened application and research value.^26^

Despite being in the early stages of exploration, the sedimentary conditions, paleoenvironmental evolution history, geochemistry, and petrophysical properties of the Fengcheng Formation in Mahu Sag have undergone extensive scrutiny.^27−29^ In recent years, the pore characteristics of Fengcheng Formation have been gradually studied. Huang identified and studied the mineral composition and pore-type characteristics of the Fengcheng Formation by X-ray diffraction (XRD), scanning electron microscopy, nuclear magnetic resonance, and other means. The comprehensive classification of pore structure was achieved through core nuclear magnetic resonance experiments and two-dimensional nuclear magnetic resonance logging, allowing for continuous evaluation of pore structure and oiliness characteristics.^30^ Li studied and analyzed shale pore structure, physical properties, and influencing factors by scanning electron microscopy, low-temperature nitrogen adsorption, high-pressure mercury injection, computed tomography (CT) scanning, etc., and explored the controlling effects of shale pore structure on shale oil potential.^31^ Different from other lacustrine shales with a higher content of organic matter and clay minerals, the contents of organic matter and clay minerals in Mahu Sag are low. Few studies have studied the pore structures of the Fengcheng Formation, particularly the relationship between minerals, lithology, and pore development. Additionally, a comparative study on the pore development characteristics of Fengcheng Formation shale samples and shale in other areas is notably lacking. This gap highlights an avenue for future research endeavors.

In this study, 113 samples from Fengcheng Formation of Well X in Mahu Sag were selected, the mineral composition of Fengcheng Formation was analyzed by XRD, and the pore characteristics of the samples were explored in combination with low-temperature nitrogen adsorption and high-pressure mercury injection. In addition, the relationship between lithology and pore development is analyzed and demonstrated by scanning electron microscopy (SEM). Finally, the samples of Fengcheng Formation from this well are analyzed and compared with those from other wells in this area and shale samples from other basins. The comprehensive examination aims to enhance the understanding of the pore development characteristics specific to the Fengcheng Formation in Mahu Sag. The insights derived from this analysis contribute novel theoretical support to inform and guide future endeavors in shale oil exploration and development within this geographical area.

## 2 Geologic Setting

**Figure 1:**
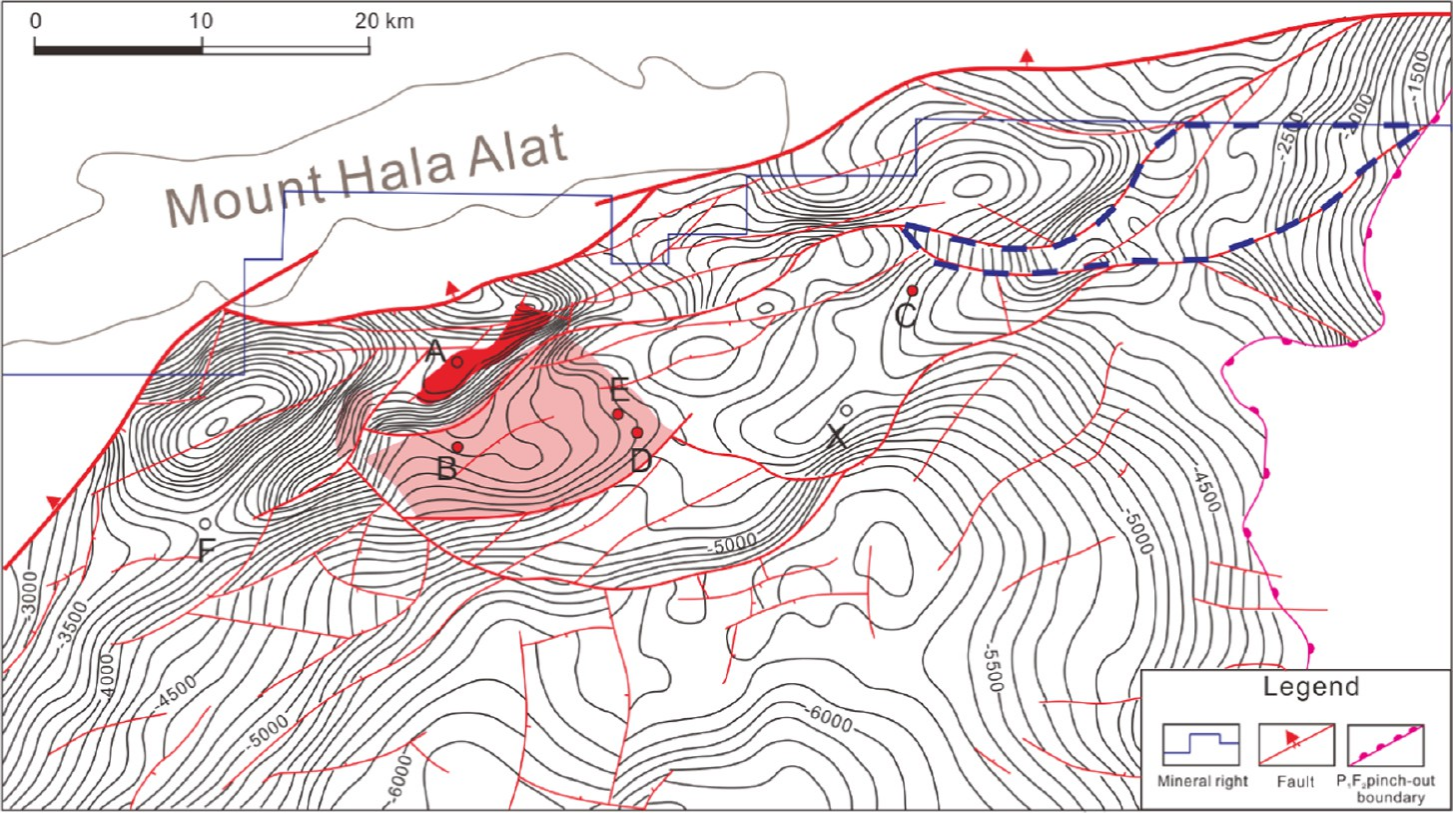
Structure contour map of Mahu Sag.

The Mahu Sag, situated in the northwestern region of the Junggar Basin as depicted in Figure 1, exhibits distinct geological features. Bounded by the Wuxia-Kebai fault belt to the west, the Zhongguai Uplift to the southwest, the Dabasong Uplift to the south, and the Xiayan Uplift, Sangquan Uplift, Yingxi Depression, and Shiyingtan Uplift to the east and northeast, the sag encompasses an area of approximately 5000 km^2^.^32−34^ Recognized as the most oil-rich sag in the Junggar Basin, the geological evolution of Mahu Sag has been influenced significantly by the collision and compression between the Western Junggar Ocean and the Kazakhstan plate. Particularly noteworthy is the collision during the Middle and late Carboniferous to early Permian, resulting in the formation of extensive nappes in the northwestern basin and the establishment of the foreland depression in the Mahu-West Sag of Pen 1.^35,36^

The development of foreland basins is often accompanied by the deposition of high-quality source rocks.^37^ In particular, the sedimentary period of the Fengcheng Formation of the Lower Permian Series was the most vigorous development period of the western foreland basin, which formed the most important set of hydrocarbon source rocks in Junggar Basin.^38,39^ Positioned beneath the Jiamuhe Formation and above the Xiazijie Formation, the Fengcheng Formation consists of three members, namely, Feng 1 Member (P_1_f_1_), Feng 2 Member (P_1_f_2_), and Feng 3 Member (P_1_f_3_). The lithofacies exhibit significant variations from the bottom to the top. Early in the Feng 1 Member, frequent volcanic activity dominated the northeastern part of the Mahu Depression, characterized by pyroclastic rocks and sedimentary pyroclastic rocks. After that, organic-rich mudstone and dolomite are developed successively. With the increasing salinity of the lake basin, a large number of alkaline minerals such as sodium, gabbro, and mica developed in the middle depression during the sedimentation of Feng 2 Member. The salinity of the lake basin in Feng 3 Member decreased, and the sag was dominated by dolomite. Terrigenous clastic rocks are developed at the top of Feng 3 Member. The closer to the foothills of Zaire, the higher the content of clastic rocks and the larger the particles. Longitudinally, the rich reed breccia developed near the deep fault of the sag.^39,40^

**Figure 2:**
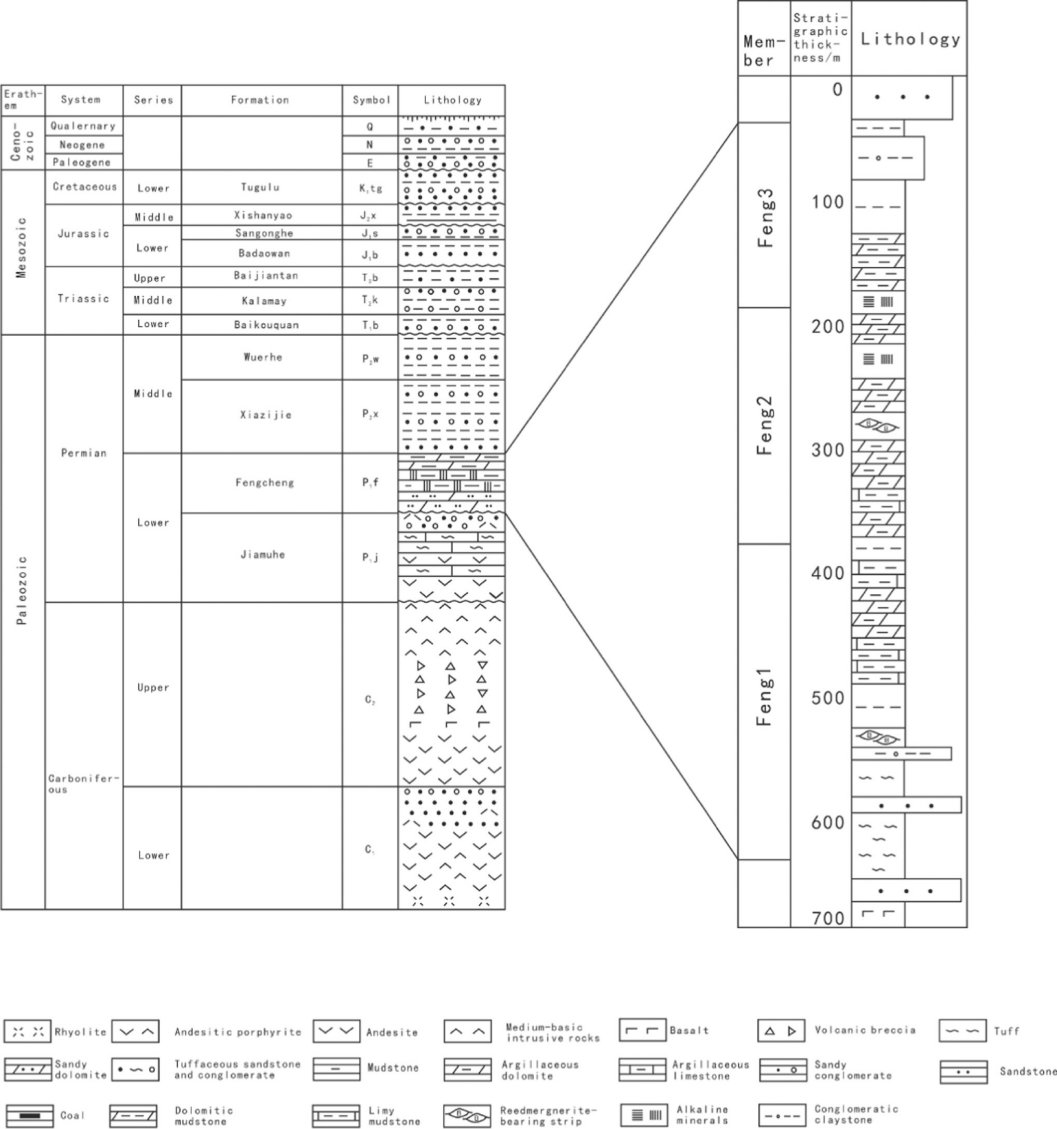
Stratigraphic column map of Junggar Basin (adapted with permission from refs. (41−44) Copyright Cao et al., Shi et al., Xiao et al., Yu et al.).

Figure 1 illustrates the primary focus well, well X, which serves as the central well for this study. Wells A, B, C, D, E, and F are strategically chosen locations for comparison samples, contributing to a comprehensive understanding of the geological characteristics within Mahu Sag (Figure 2).

## 3 Samples and Methods

### 3.1 Samples

This study focused on the analysis of 113 samples extracted from Well X within the Fengcheng Formation Feng 2 member in Mahu Sag, located in the Junggar Basin, Xinjiang. These samples were obtained from cores at various depths. All 113 samples underwent X-ray diffraction (XRD) and low-temperature nitrogen adsorption experiments. Subsequently, based on the mineral composition results derived from XRD, 49 samples were specifically chosen for high-pressure mercury injection experiments. Finally, the outcomes of both the high-pressure mercury injection experiment and XRD mineral composition analysis guided the selection of a refined subset of 20 samples for further examination through scanning electron microscopy (SEM). This systematic approach ensures a comprehensive and targeted analysis of the geological and pore structure characteristics within the Fengcheng Formation in Mahu Sag.

### 3.2 Methods

#### 3.2.1 X-ray Diffraction (XRD)

In this study, X-ray diffraction (XRD) experiments were conducted using the RINT-TTR3 X-ray diffractometer, adhering to the guidelines outlined in the China Petroleum Industry Standard SY/T 5163–2018. The experimental procedure involved grinding the sample into a 200 mg powder. Cu Kα radiation was employed, with a scanning speed of 2°/min, a sampling step width of 0.02°, and a scanning range from 5 to 45°. X-ray diffraction spectra were generated, and semiquantitative calculations of the mineral composition were performed based on the peak areas corresponding to each mineral on the X-ray spectra.

#### 3.2.2 Low-Temperature Nitrogen Adsorption (LTNA)

In this study, 113 samples were ground to 60 mesh powder. About 2 g of each sample was taken and degassed at 110 °C in the Micromeritics Smart Vacprep degassing instrument for 12 h. Then, the Micromeritics TriStar II Plus 3030 instrument was used for the nitrogen adsorption experiment at 77 K. According to the Brunauer–Emmett–Teller (BET) method,^45^ the specific surface area of the shale pores can be obtained. The pore volumes of different pore sizes and mean pore sizes can be obtained by the Barrett–Joyner–Halenda (BJH) model.^46,47^

In this investigation, the fractal dimension of low-temperature nitrogen adsorption (LTNA) was computed utilizing the FHH fractal model.^48−53^ The analysis distinguished two segments: the low-pressure segment (*p*/*p*_0_ < 0.5, denoted as *D*_1_), primarily influenced by van der Waals forces and characterized by pore surface features. A larger *D*_1_ signifies a more irregular internal pore surface shape, indicating decreased smoothness. *D*_1_ significantly impacts adsorption performance and is predominantly controlled by micropores. In contrast, the high-pressure section (*p*/*p*_0_ > 0.5, denoted as *D*_2_) is governed by interfacial tension, with *D*_2_ reflecting pore structure characteristics, such as distribution and size. A larger *D*_2_ indicates dispersed pore distribution and smaller pore sizes, exerting greater influence on gas seepage and specific pore volume. *D*_2_ is predominantly influenced by clay minerals content and thermal maturity.^54,55^

FHH calculates the fractal dimension with the following formula:
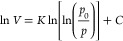
1
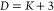
2where *p*_0_ is the saturated steam pressure, *p* is the equilibrium pressure, Mpa; *V* is the adsorption amount of nitrogen when the pressure is *p*, equivalent to the pore volume, m^3^; *D* is the fractal dimension; and *K* and *C* are constants of the function.

When the fractal dimension is small, the pore space development is relatively simple, the distribution of pore size and pore volume is concentrated, the heterogeneity is weak, and the pore connectivity is good. When the fractal dimension is large, the pore morphology is complex and varied, the pore size and pore volume distribution are dispersed, the heterogeneity is strong, and the pore connectivity is poor. Fractal dimension *D* is generally between 2 and 3, and when it is close to 2, it indicates that the pore surface is smooth and the pore connectivity is good. When it is close to 3, the pore surface is rough, the heterogeneity is strong, and the pore connectivity is very poor.

The configuration of the low-temperature nitrogen adsorption–desorption curve provides valuable insights into the pore structure characteristics of shale reservoirs. The fundamental principle underlying this analysis is the observation of adsorption hysteresis in shale, where the adsorption curve and desorption curve exhibit separation, resulting in the formation of a hysteresis loop. The characteristics of this loop are indicative of the shale’s pore shape, allowing for inferences regarding its pore structure.^56^ To delineate aperture sizes, the IUPAC partitioning scheme is commonly employed.^56^ This classification categorizes pores into micropores, mesopores, and macropores, with micropores having a size less than 2 nm, mesopores ranging from 2 to 50 nm, and macropores exceeding 50 nm. This standardized approach facilitates a systematic and precise characterization of the shale’s pore size distribution, contributing to a comprehensive understanding of its structural properties.

#### 3.2.3 High-Pressure Mercury Intrusion (HPMI)

In this study, the American AutoPore IV 9500 mercury injection instrument served as the experimental tool for high-pressure mercury injection. Preceding the experiments, samples underwent a drying process until a constant weight was achieved at 105 °C. The maximum experimental pressure for the mercury injection test was set at 200 MPa. The experimental procedures strictly adhered to the national standards of the People’s Republic of China, specifically GBT 29172–2012 “Core Analysis Method” and GB/T 29171–2012 “Determination of Rock Capillary Pressure Curve”. The high-pressure mercury injection (HPMI) experiment facilitated the determination of crucial radius parameters such as porosity, permeability, and average porosity. The fractal dimension of mercury injection is calculated by the following formula:^57,58^
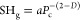
3where *a* is a constant, the formula generally shows a log–log harmonious linear relationship between capillary pressure (*P*_c_) and mercury saturation (SH_g_) (log(SH_g_)–log(*P*_c_)). The fractal dimension can then be derived from the slope of the line.^59^

#### 3.2.4 Field Emission Scanning Electron Microscopy (FE-SEM)

This study employed the American Quanta FEG 650 for field emission scanning electron microscope (FE-SEM) experiments, which was equipped with an energy dispersion spectrometer for mineral identification. To enhance the portrayal of pore morphology, the shale surface underwent preparation using the Gatan argon-ion cross section polishing machine, manufactured in the United States, before the imaging observation. Subsequent to carbon plating, FE-SEM experiments were conducted at a voltage of 20 kV and a working distance of 9.8 mm. This methodological approach ensures a comprehensive and detailed examination of the shale surface, incorporating mineral identification and emphasizing pore characteristics for a thorough analysis.

## 4 Results

### 4.1 Petrologic Feature

**Figure 3:**
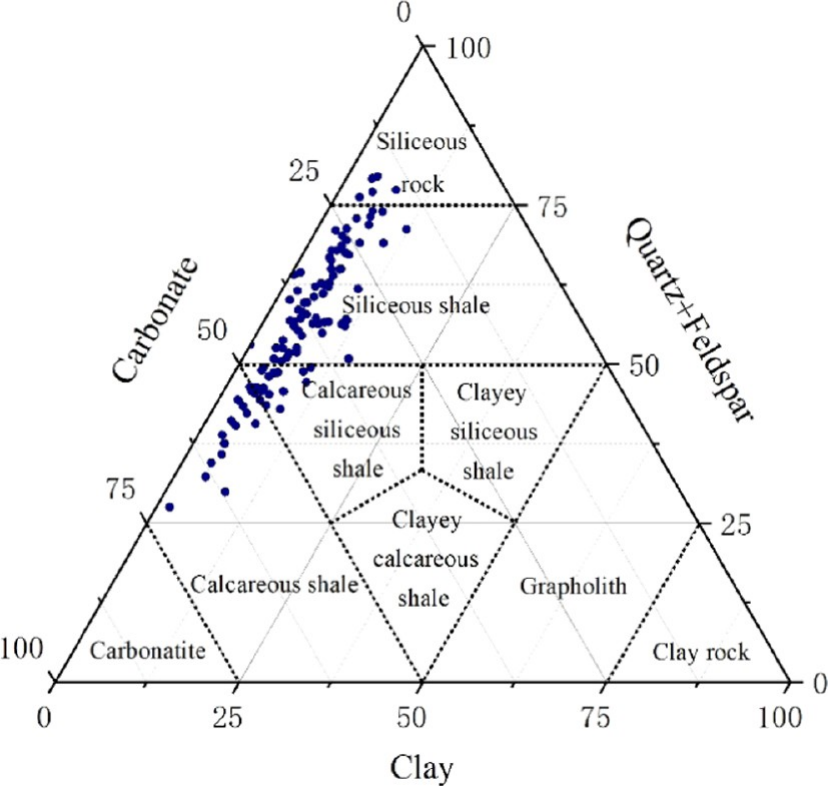
Ternary lithofacies map of Mahu shale.

The results of X-ray diffraction (XRD) quantitative analysis reveal significant variations in mineral composition among samples from the Fengcheng Formation shale in Mahu Sag. The majority of samples exhibit high quartz and plagioclase content, with quartz ranging from 1 to 76.8%, averaging 27.6%, and plagioclase ranging from 1.8 to 55.7%, averaging 21.5%. Additionally, certain samples demonstrate elevated levels of carbonate minerals, including calcite (0.3 to 63.3%, averaging 14.9%) and dolomite (1.6 to 60.4%, averaging 21.3%). In contrast, clay mineral content is relatively low, ranging from 0.4 to 19.4%, with an average of 5.4%. These findings underscore the diverse mineralogical composition and lithological complexity of the Fengcheng shale. The shale lithofacies ternary map (Lithofacies classification is based on^61,62^) of Fengcheng Formation in Mahu Sag shows that the lithology is mainly composed of siliceous shale, calcareous siliceous mixed shale, and calcareous shale, while the content of siliceous rock is relatively small. This conclusion is consistent with previous research conclusions.^32,60,61^ These comprehensive mineralogical insights contribute to a nuanced understanding of the geological characteristics of the Fengcheng Formation in Mahu Sag (Figure 3).

### 4.2 Pore Characteristics Analyzed by LTNA

According to the LTNA experiment, a total of 6 types of pore parameters were obtained in this paper, which were respectively hysteresis loop area, specific surface area, pore volume, mean pore size, pore surface fractal dimension, pore structure, and fractal dimension. The specific surface area of the samples from Mahu Fengcheng Formation ranged from 0.2738 to 7.9956 m^2^/g, with an average value of 2.0895 m^2^/g. The pore volume ranges from 0.0005810 to 0.01526 mL/g, with an average value of 0.004590 mL/g. The mean pore size was from 11.2758 to 35.8561 nm, with an average of 25.2019 nm. According to the lithology analysis of the lithofacies ternary map, the pore structure of different lithologies is analyzed in depth. The results revealed that due to the limited quantity of siliceous rocks and the absence of statistically significant data patterns, the pore structure of siliceous rock samples was not included in the current study. This nuanced approach ensures a focused exploration of pore characteristics in relation to lithological variations within the Mahu Fengcheng Formation, providing a comprehensive and targeted understanding of the shale reservoir’s pore structure.

**Figure 4:**
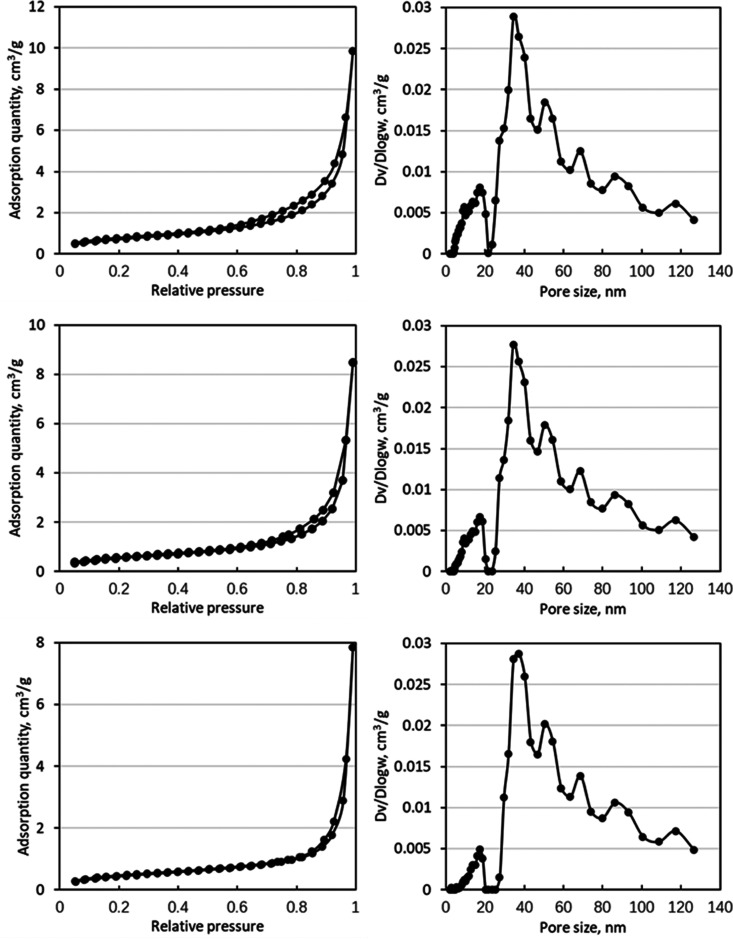
Images of adsorption and desorption and pore size distribution of typical samples

Figure 4 shows the adsorption and desorption curves and pore size distributions for some typical samples. The adsorption and desorption curves of the samples exhibit striking similarities, resembling typical Type IV adsorption isotherms.^63^ The hysteresis loop observed is narrow, indicating the occurrence of capillary condensation under saturated vapor pressure conditions. The shape of the hysteresis loop indicates the existence of the slit-shaped pores in these samples. Regarding the pore size distribution, the analysis primarily reveals a bimodal distribution. The graph displays a prominent peak within the 0–20 nm range, followed by a higher peak spanning 20–40 nm.

**Figure 5:**
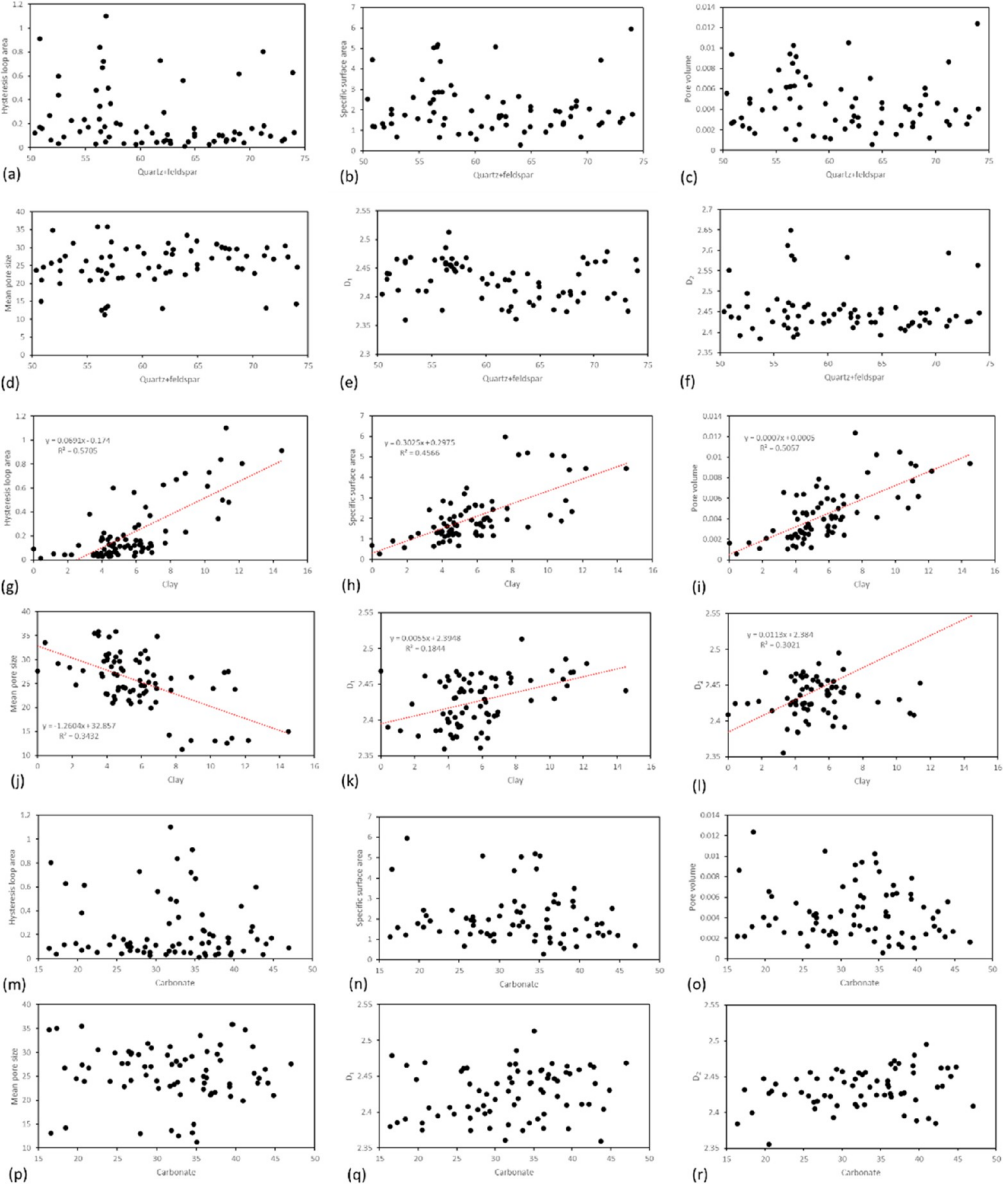
Relationship between different mineral contents and nitrogen adsorption parameters in siliceous shale. (a–f) Relationship between quartz + feldspar content and hysteresis loop area, specific surface area, pore volume, mean pore size, *D*_1_, and *D*_2_. (g–l) Relationship between clay mineral content and hysteresis loop area, specific surface area, pore volume, mean pore size, *D*_1_, and *D*_2_. (m–r) Relationship between carbonate content and hysteresis loop area, specific surface area, pore volume, mean pore size, *D*_1_, and *D*_2_.

Figure 5 illustrates the correlation between the contents of quartz + feldspar, clay minerals, and carbonate within siliceous shale and various pore parameters, including hysteresis loop area, specific surface area, pore volume, mean pore size, *D*_1_, and *D*_2_. The analysis reveals that there is no discernible relationship between quartz + feldspar and carbonate content with any of the considered parameters. In contrast, clay minerals exhibit a positive correlation with hysteresis loop area, specific surface area, pore volume, *D*_1_, and *D*_2_. Conversely, clay minerals show a negative correlation with mean pore size. These findings contribute to a nuanced understanding of the influence of mineral composition, specifically clay minerals, on the pore characteristics of siliceous shale within the Mahu Fengcheng Formation.

#### 4.2.1 Calcareous Siliceous Shale

**Figure 6:**
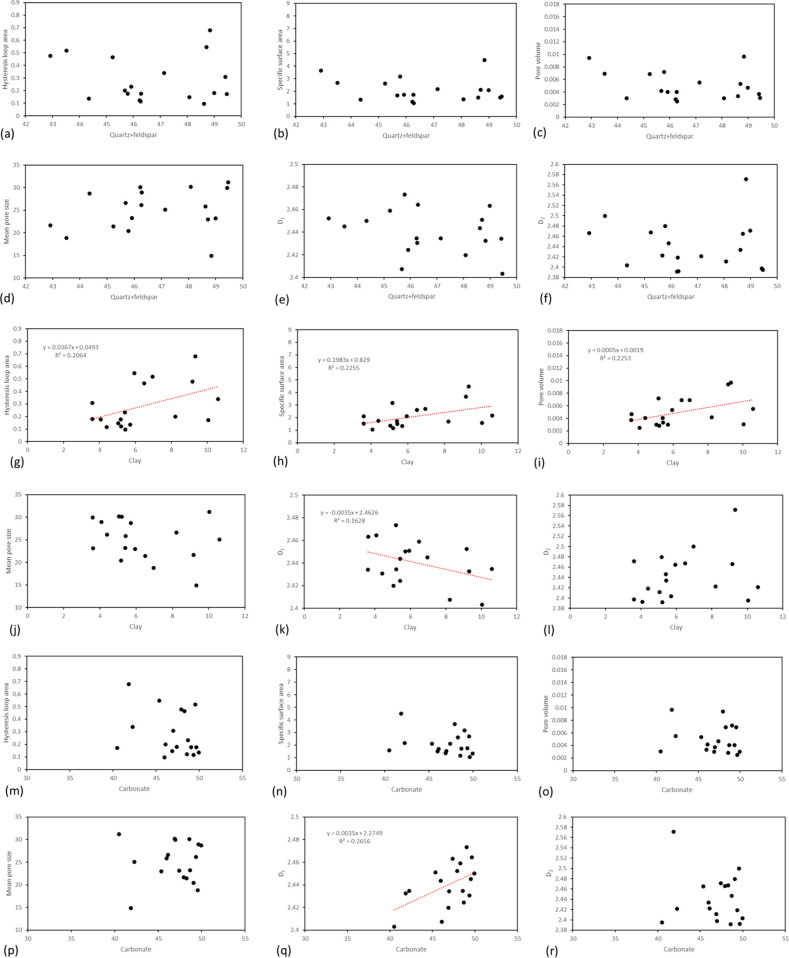
Relationship between different mineral contents and nitrogen adsorption parameters in calcareous siliceous shale. (a–f) Relationship between quartz + feldspar content and hysteresis loop area, specific surface area, pore volume, mean pore size, *D*_1_, and *D*_2_. (g–l) Relationship between clay mineral content and hysteresis loop area, specific surface area, pore volume, mean pore size, *D*_1_, and *D*_2_. (m–r) Relationship between carbonate content and hysteresis loop area, specific surface area, pore volume, mean pore size, *D*_1_, and *D*_2_.

Figure 6 depicts the relationship between the contents of quartz + feldspar, clay minerals, and carbonate in calcareous siliceous shale and various pore parameters, including hysteresis loop area, specific surface area, pore volume, mean pore size, *D*_1_, and *D*_2_. Notably, no discernible relationship is observed between quartz + feldspar and any of the investigated parameters. In contrast, clay minerals exhibit a positive linear correlation with hysteresis loop area, specific surface area, and pore volume, while demonstrating a negative linear correlation with *D*_1_. However, the correlation coefficients for these relationships are comparatively lower than those observed in the analysis of siliceous shale. Furthermore, carbonate rocks show a positive correlation with *D*_1_, although the correlation coefficient is relatively low. These findings provide nuanced insights into the varying impact of mineral composition on pore characteristics within calcareous siliceous shale, highlighting differences from the patterns observed in siliceous shale within the Mahu Fengcheng Formation.

#### 4.2.2 Calcareous Shale

**Figure 7:**
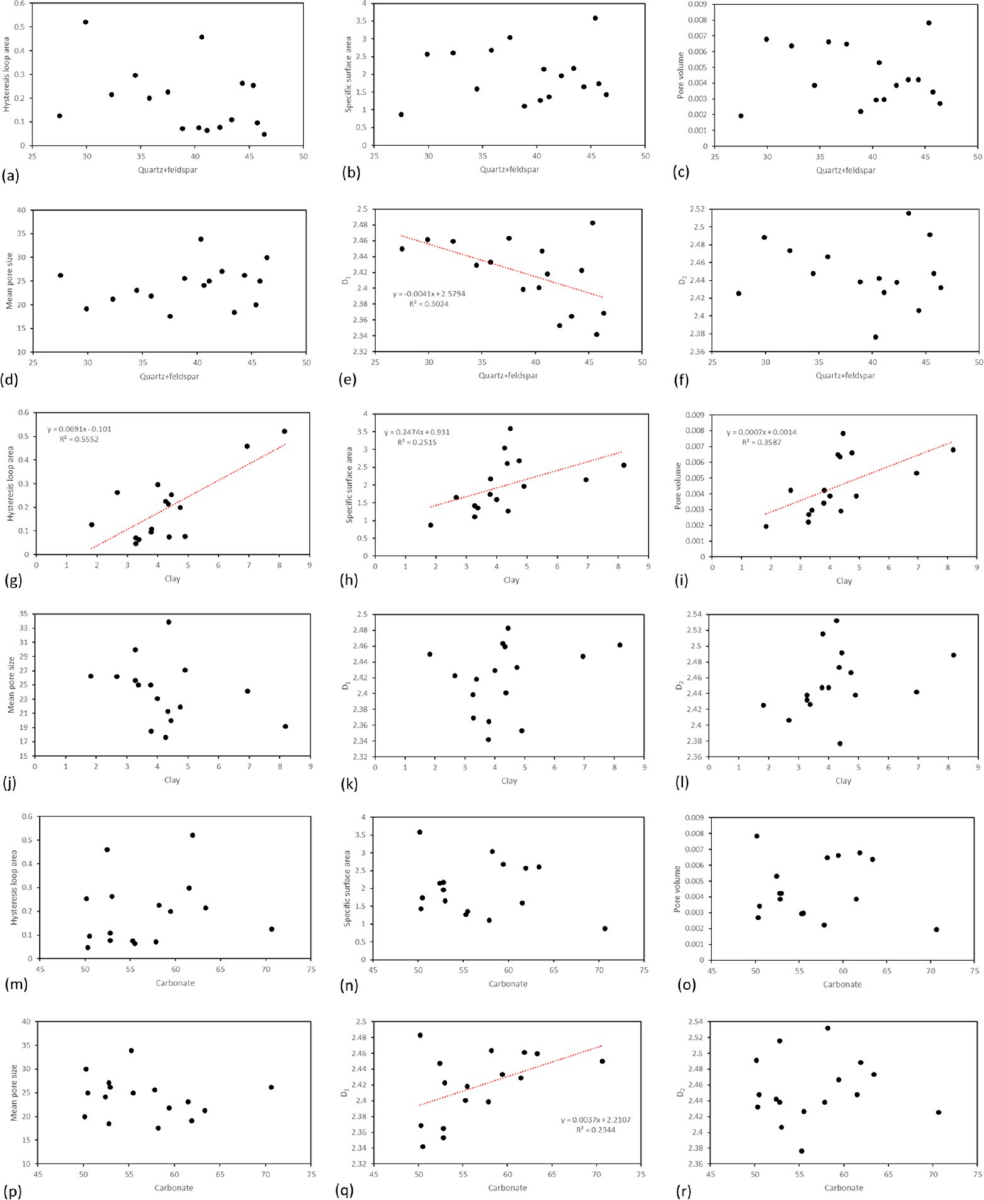
Relationship between different mineral contents and nitrogen adsorption parameters in calcareous shale. (a–f) Relationship between quartz + feldspar content and hysteresis loop area, specific surface area, pore volume, mean pore size, *D*_1_, and *D*_2_. (g–l) Relationship between clay mineral content and hysteresis loop area, specific surface area, pore volume, mean pore size, *D*_1_, and *D*_2_. (m–r) Relationship between carbonate content and hysteresis loop area, specific surface area, pore volume, mean pore size, *D*_1_, and *D*_2_.

As depicted in Figure 7, within calcareous shale, quartz + feldspar displays a linear negative correlation specifically with *D*_1_ and exhibits no evident relationship with other investigated parameters. Conversely, clay minerals show positive correlations with hysteresis loop area, specific surface area, and pore volume, while displaying no discernible relationships with other parameters. Notably, a linear positive correlation is observed between carbonate and *D*_1_, although no apparent relationships are evident between carbonate and other investigated parameters. These observations underscore the intricate interplay between mineral composition and pore characteristics within calcareous shale, with specific minerals demonstrating distinct correlations with certain pore parameters.

The analysis results of different lithology and all samples are shown in Table 1. It can be seen that although the clay mineral content of Fengcheng Formation is not high, it is extremely related to the development of pores.

**Table 1:** Relationship between Minerals and Nitrogen Adsorption Pore Parameters in Different Lithology^a^

		pore parameter					
lithology	mineral composition	hysteresis loop area	specific surface area	pore volume	mean pore size	*D*_1_	*D*_2_
siliceous shale	quartz + feldspar						
	clay	P	P	P	N	P	P
	carbonate						
calcareous siliceous shale	quartz + feldspar						
	clay	P	P	P		N	
	carbonate					P	
calcareous shale	quartz + feldspar					N	
	clay	P	P	P			
	carbonate					P	

a Note: P means linear positive correlation, N means linear negative correlation.

Table 1 presents the analysis results for various lithologies and all samples. Despite the relatively low clay mineral content in the Fengcheng Formation, a significant correlation emerges between clay minerals and pore development. This observation underscores the influential role of clay minerals in shaping the pore characteristics within the formation.

### 4.3 Pore Characteristics Analyzed by HPMI

**Figure 8:**
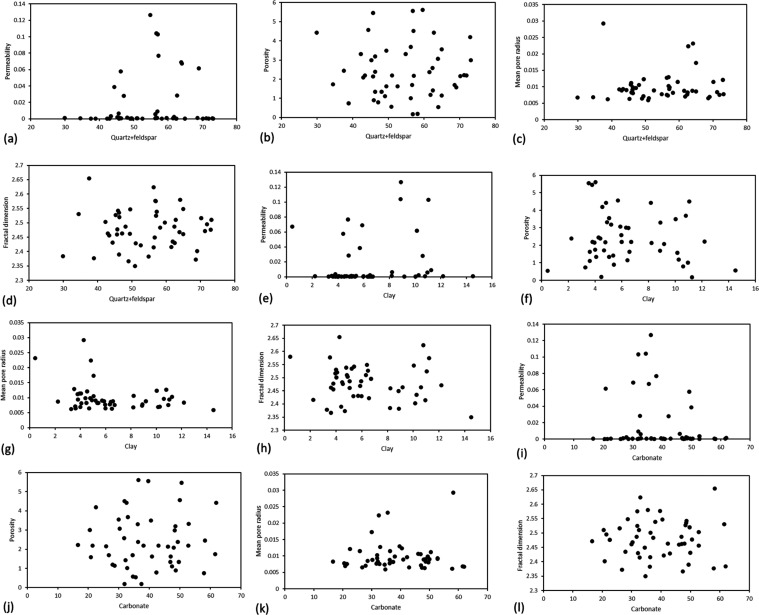
Relationship between different mineral contents and mercury injection parameters of all samples. (a–d) Relationship between quartz + feldspar content and permeability, porosity, mean pore radius, and fractal dimension; (e–h) Relationship between clay minerals content and permeability, porosity, mean pore radius, and fractal dimension. (i–l) Relationship between carbonate content and permeability, porosity, mean pore radius, and fractal dimension.

In this paper, 49 samples were selected for high-pressure mercury injection experiment. Figure 7 illustrates the relationship between the content of various minerals and key reservoir parameters, including porosity, permeability, mean pore radius, and fractal dimension. Permeability ranges from 0.000146 to 0.1266 mD with an average value of 0.016521 mD, while porosity ranges from 0.181 to 5.61% with an average value of 2.407%. These findings indicate that the reservoir of Fengcheng Formation is characterized by low porosity and low permeability. The mean pore radius ranges from 0.00586 to 0.02924 μm, with an average value of 0.00917 μm. The fractal dimension ranges from 2.3494 to 2.6549, with an average value of 2.4779. As shown in Figure 8, the content of the three minerals has no obvious relationship with permeability, porosity, mean pore radius, and fractal dimension.

**Figure 9:**
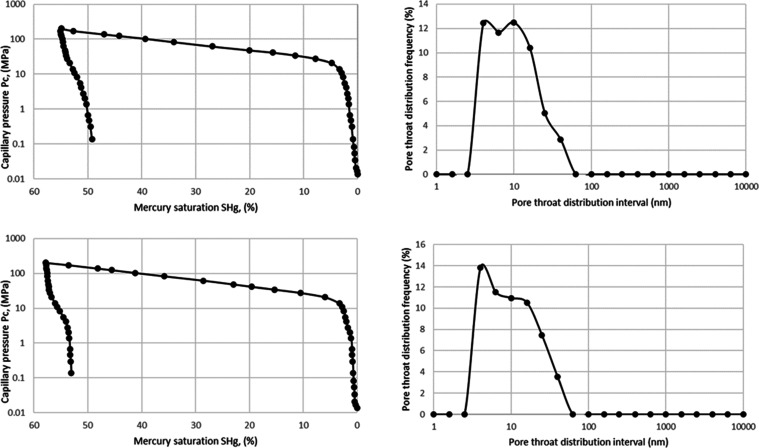
Capillary pressure curve and pore throat distribution of typical samples.

Figure 9 shows the capillary pressure curve and pore throat distribution of some samples by using the mercury intrusion method. The capillary pressure curves for all samples exhibit a similar trend, characterized by a rapid rise in capillary pressure upon mercury saturation below 5%, followed by a gradual increase until reaching maximum saturation. In terms of pore throat distribution, a single peak is evident with a peak value of approximately 10 nm. Remarkably, the pore throat distribution acquired through mercury injection closely aligns with that obtained via nitrogen adsorption, indicating consistency between the two methods.

### 4.4 Pore Characteristics Observed by FE-SEM

**Figure 10:**
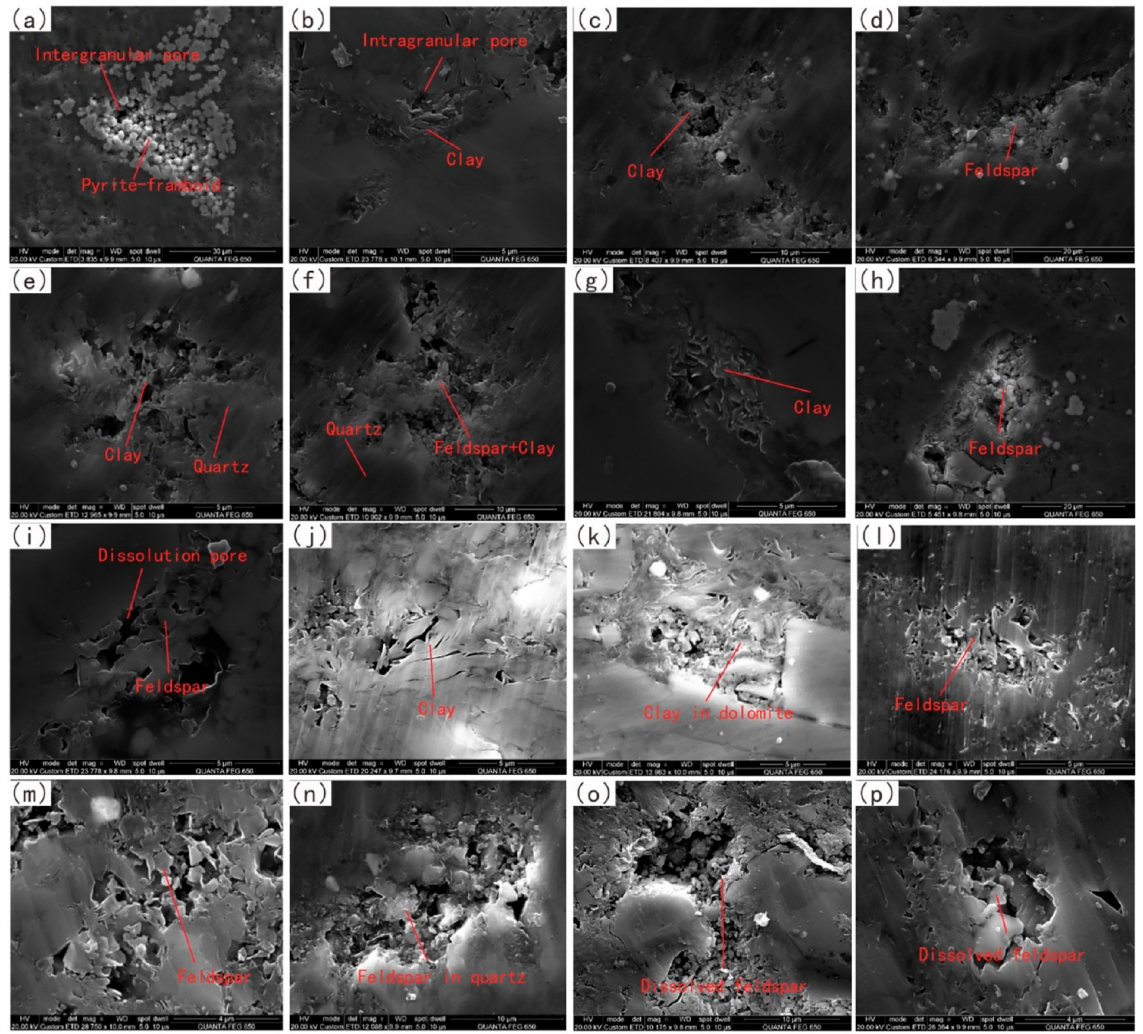
FE-SEM experiment image of well X. (a) Intergranular pores developed in pyrite-framboid; (b) intergranular pores developed in clay minerals; (c) development of larger intragranular pores in clay minerals; (d) pores developed around feldspar; (e) pores developed around clay minerals in the quartz matrix; (f) pores developed around clay minerals and feldspar in the quartz matrix; (g) intergranular pores developed in clay minerals; (h) large pores developed in feldspar; (i) dissolved pores developed in feldspar; (j) strip pores developed in clay minerals; (k) pores developed around clay minerals in the dolomite matrix; (l) intragranular pores developed in feldspar; (m) intergranular pores developed in feldspar; (n) pores developed around feldspar in quartz matrix; (o) pores resulting from the dissolution of feldspar; and (p) pores created by the dissolution of feldspar in the dolomite matrix.

According to the classification of Loucks,^9^ pores in shale can be divided into intragranular pores, intergranular pores, and organic pores. The organic matter content of this batch of samples is low, with most TOC content less than 1%. The organic matter and organic pores are rarely observed under the microscope. Therefore, organic pores in Fengcheng Formation are less developed and inorganic pores are mainly developed. Intergranular pores (Figure 10a) can be seen in pyrite-framboid, and most of the remaining pores are intragranular pores, which are mostly developed around feldspar and clay minerals. The dissolution of feldspar is evident in the formation of pores, either autonomously on its own as a substrate (Figure 10d,i,l,o) or on substrates such as quartz or calcite (Figure 10n,p). Furthermore, feldspar can undergo conversion to clay minerals, with pores also forming during this transformative process (Figure 10f).

## 5 Discussion

### 5.1 Influence of Minerals on Pore Development

In this investigation, the nitrogen adsorption results indicate a pronounced association between pore parameters and clay minerals, with minimal correlation observed with quartz + feldspar and carbonate. Examination of FE-SEM images reveals that pores predominantly manifest around clay minerals and feldspar. XRD results further corroborate these findings, indicating that although the clay mineral content in well X samples is not substantial, the feldspar content is notably high.

In the context of conventional oil and gas exploration, prior research by scholars has demonstrated that the thermal evolution of source rocks can liberate significant quantities of short-chain organic acids. These acids, in turn, facilitate the dissolution of numerous feldspar and carbonate minerals, thereby creating ample reservoir-level pore scales.^64^ Following fluid–rock interactions, the content of feldspar minerals tends to decrease with increasing thermal evolution, while the content of clay minerals and carbonate minerals rises. Conversely, the content of quartz remains relatively unchanged.^65^ Thus, the fluid generated during the oil and gas evolution of source rocks induces the dissolution of feldspar minerals in feldspar sandstone reservoirs, resulting in the formation of clay minerals and pores.

The observed pores in the FE-SEM images predominantly appear around feldspar and clay minerals, with pores generated through feldspar dissolution being more prevalent. This observation substantiates the notion that the fluid–rock interaction during the evolution of oil and gas from source rocks leads to the dissolution of feldspar minerals in the feldspar sandstone reservoir, subsequently giving rise to clay minerals and pores. Despite the relatively low clay content in the Fengcheng Formation, the high prevalence of feldspar, often dissolved to form pores and clay minerals, aligns with the conclusions drawn from the nitrogen adsorption experiment.

**Figure 11:**
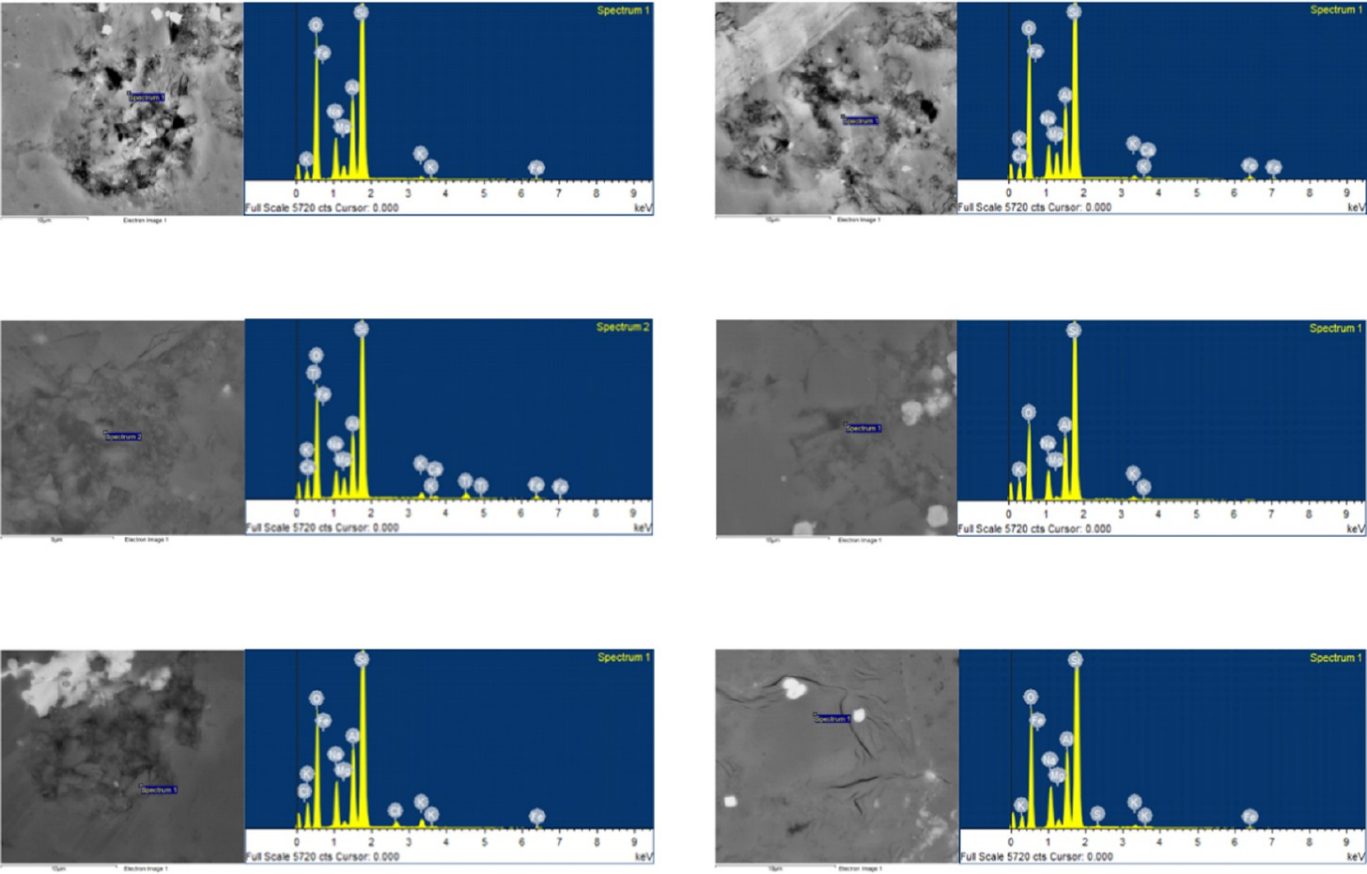
Plagioclase in SEM backscattering images of well X and EDS spectra.

A predominant feature of the samples from Well X is the elevated content of plagioclase, coupled with a comparatively low content of potassium feldspar. This observation is further substantiated by the abundance of plagioclase evident in the FE-SEM images (Figure 11). The conversion processes between feldspar and clay minerals, such as potassium feldspar and plagioclase, yield kaolinite as the dissolution product in potassium-poor acidic fluids and Illite as the dissolution product in potassium-rich acidic fluids. This chemical transformation is elucidated by the following reactions.^66−68^
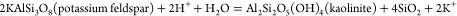
4
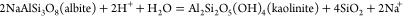
5
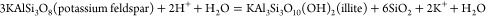
6
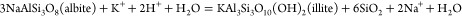
7

### 5.2 Comparison with Other Wells in the Same Area

**Figure 12:**
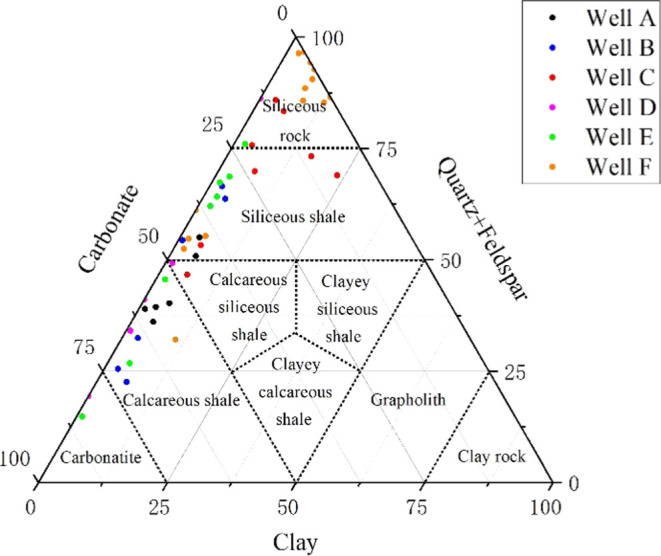
Ternary lithofacies maps of other 6 wells of Fengcheng Formation in Mahu Sag.

In this study, six additional wells (Wells A, B, C, D, E, and F) situated in the same geographic area as Well X were chosen for comparative analysis. The locations of these wells are illustrated in Figure 1, the petrographic division is depicted in Figure 12, comparison of nitrogen parameters is shown in Figure 11, and the mineral composition and nitrogen adsorption parameters are analyzed: Relationship between different mineral contents and nitrogen adsorption parameters in well A–F. The lithofacies composition of these six wells closely resembles that of Well X, predominantly featuring siliceous rock, siliceous shale, calcareous siliceous shale, and calcareous shale.

**Figure 13:**
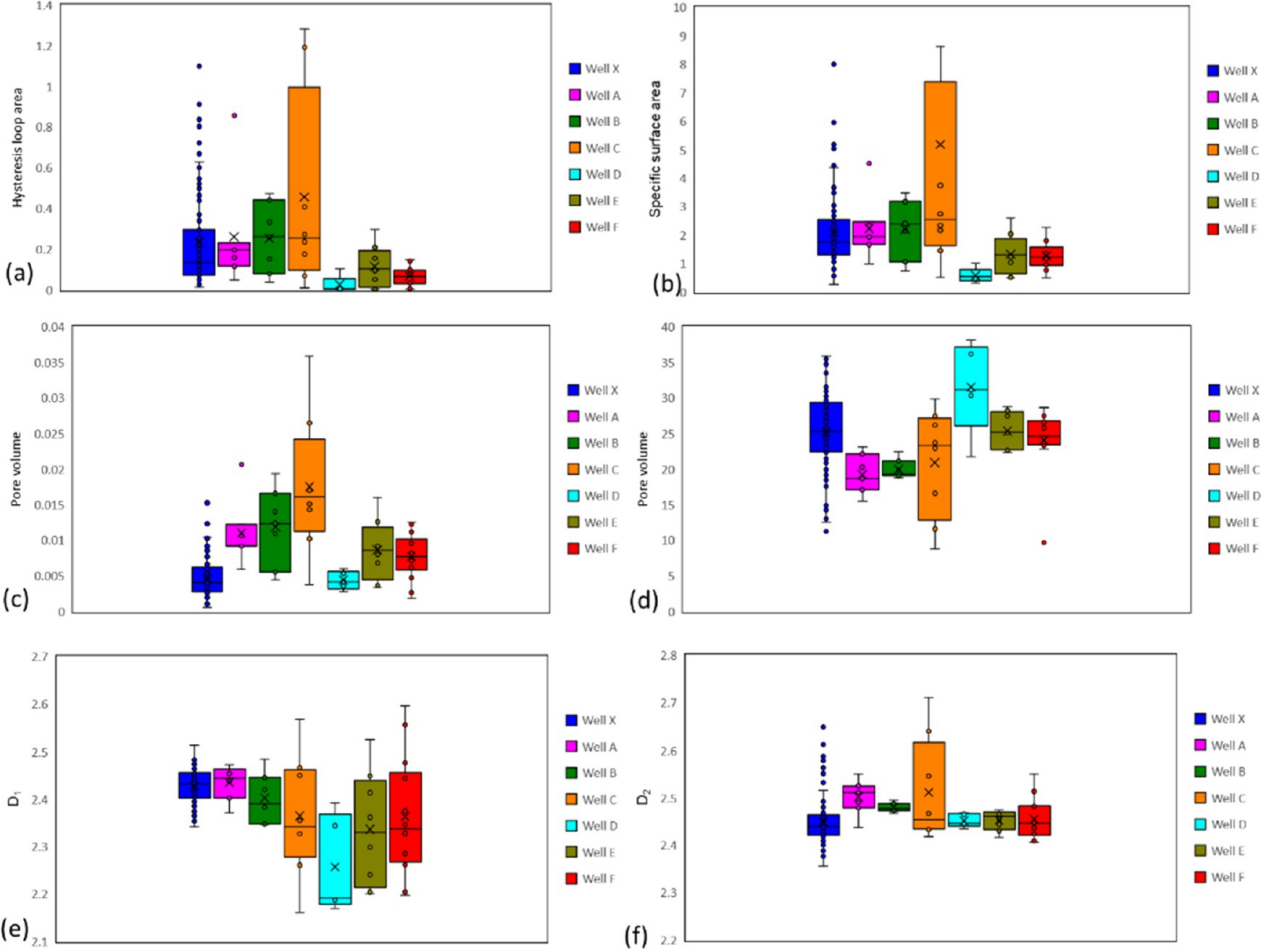
Comparison of nitrogen adsorption parameters of 7 wells of Fengcheng Formation in Mahu Sag: (a) comparison of hysteresis loop area; (b) comparison diagram of specific surface area; (c) comparison diagram of pore volume; (d) comparison diagram of mean pore size; (e) comparison diagram of *D*_1_; (f); comparison diagram of *D*_2_.

As can be seen from Figure 13, well C has relatively large hysteresis loop area, specific surface area, and pore volume, while well D has relatively small hysteresis loop area, specific surface area, and pore volume, while the mean pore size is large. The distribution range of nitrogen adsorption data of these 7 wells is similar, indicating that the pore structure development of Fengcheng Formation is similar. Upon examination of the mineral composition and nitrogen adsorption analysis diagrams, it is evident that the findings for Wells A, B, C, D, and E align with those of Well X, revealing a significant correlation between clay mineral content and nitrogen adsorption parameters. However, Well F deviates from this pattern, exhibiting no apparent relationship between clay mineral content and nitrogen adsorption parameters. Instead, a potential linear relationship is observed between quartz + feldspar in Well F, distinguishing it from the other wells. It is noteworthy that Well F is centrally located within the sag, while the other wells are situated at the sag’s periphery. Consequently, the mineral composition of Well F differs markedly from that of the other wells. Well F samples exhibit a notably high content of quartz + feldspar, primarily consisting of siliceous rocks. In contrast, samples from the other wells are composed of calcareous shale, siliceous shale, and calcareous siliceous shale. Therefore, the nitrogen adsorption parameters in Well F display a strong correlation with quartz + feldspar. Conversely, the samples from the other wells exhibit minimal siliceous rock composition, with only Wells X, C, and E containing a small amount of siliceous rock. Some of the potassium feldspar and albite in the sedimentary center of Fengcheng Formation are disordered, while the anorthite and albite in the marginal area are ordered. The order degree of feldspar is mainly related to temperature, rock age, shear stress, etc. Generally, high-temperature (magmatic system) feldspar shows disordered structure, low-temperature feldspar shows ordered structure, and the order degree of feldspar in the older age is higher than that in the new age.^69^ The ordered feldspar in the lake margin area of Fengcheng Formation is mainly of clastic origin, which indicates that the feldspar in the rocks (mainly volcanic rocks) in the surrounding provenance area has been transformed into an ordered structure. The formation of disordered feldspar in the depositional center may be related to alkaline water.^70^ This discrepancy may account for Well F’s deviation from the conclusions drawn for Well X.

### 5.3 Comparison with Samples from Other Basins

**Figure 14:**
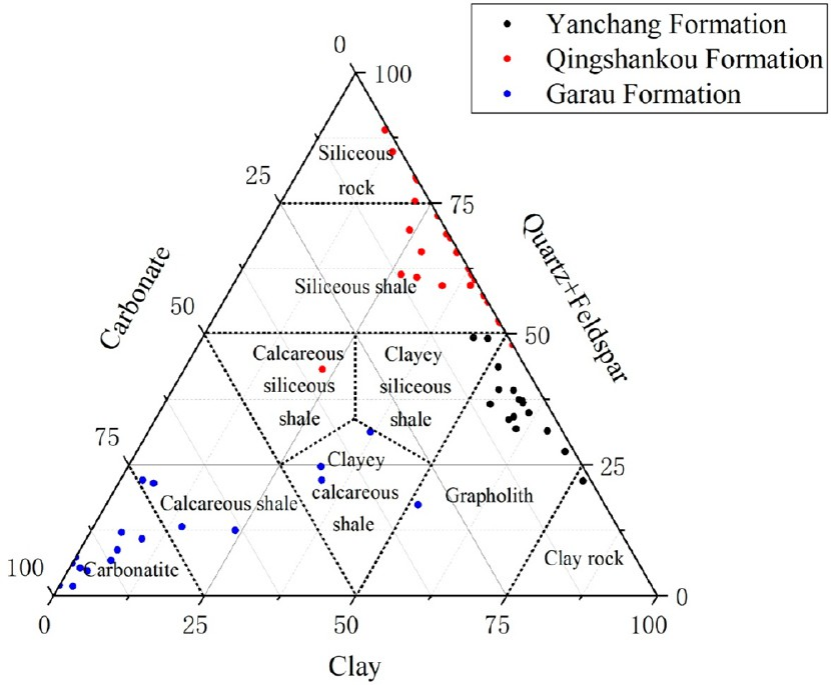
Ternary lithofacies maps in other 3 regions.

This study also includes a comparative analysis of shale samples from different regions, namely, the Yanchang Formation shale in the Ordos Basin, the Qingshankou Formation shale in the Songliao Basin, and the Garau Formation shale in the Qalikuh Locality. As illustrated in Figure 14, the lithofacies composition of these three areas significantly differs from that of the Fengcheng Formation. The Yanchang Formation is predominantly composed of clay rock and grapholith, the Qingshankou Formation consists mainly of siliceous rock and siliceous shale, while the Garau Formation is primarily composed of calcareous rock and calcareous shale.

**Figure 15:**
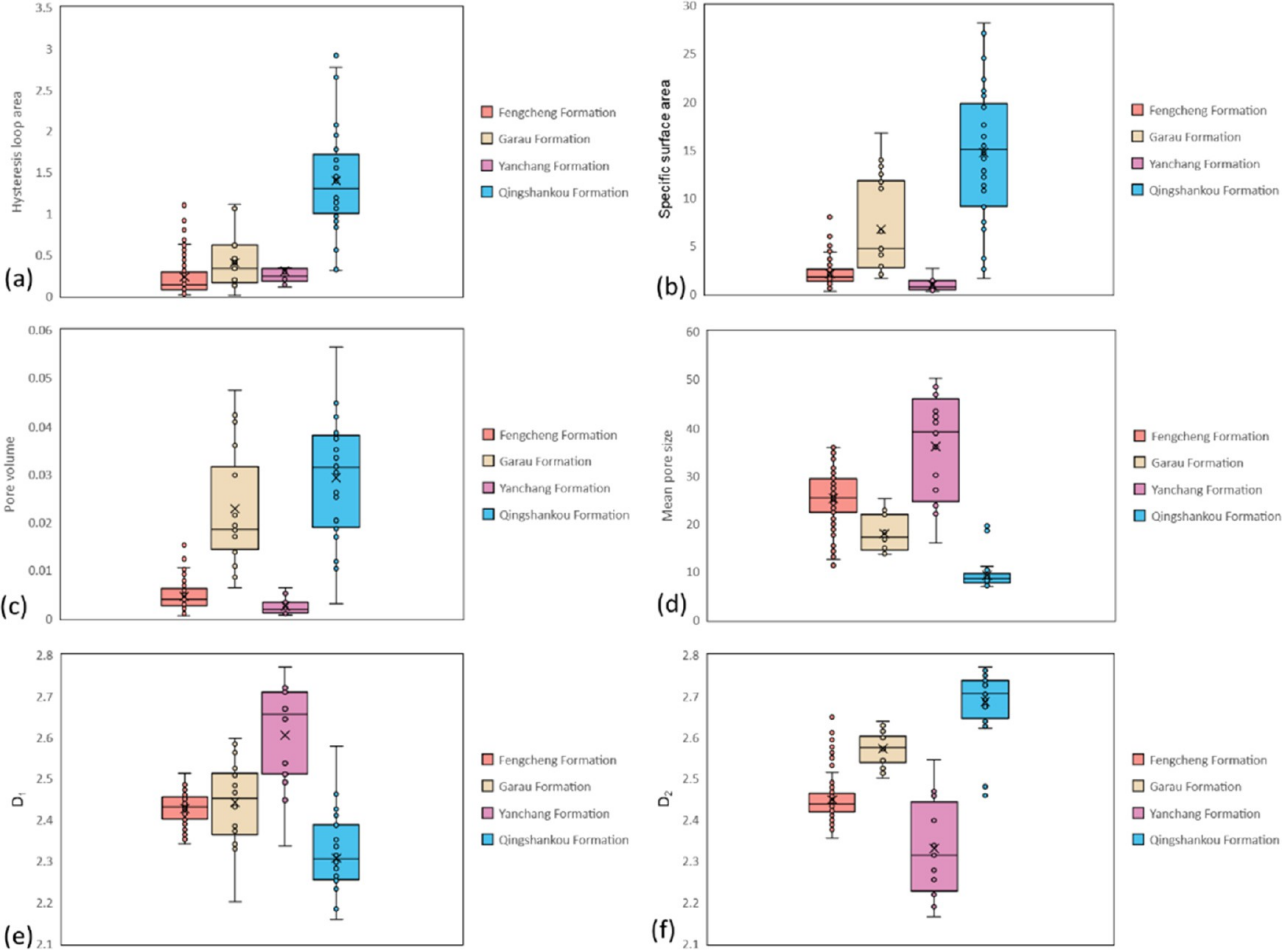
Comparison of nitrogen adsorption parameters in 4 regions: (a) comparison of hysteresis loop area; (b) comparison diagram of specific surface area; (c), comparison diagram of pore volume; (d) comparison diagram of mean pore size; (e) comparison diagram of *D*_1_; (f) comparison diagram of *D*_2_.

Analysis of nitrogen adsorption parameters depicted in Figure 15 reveals distinct characteristics among these formations. Notably, the Qingshankou Formation shale exhibits a higher hysteresis loop area, specific surface area, and pore volume, coupled with a lower mean pore size. Both Fengcheng Formation and Yanchang Formation have lower hysteresis loop area, specific surface area, and pore volume. Yanchang Formation shale exhibits the highest *D*_1_ but the lowest *D*_2_. Further examination of mineral composition and nitrogen adsorption parameters in these three areas is detailed in Table 2. In the Yanchang Formation, no consistent correlation is observed between mineral composition and nitrogen adsorption parameters, suggesting that pore development in this formation is not strongly associated with clay minerals. Conversely, in the other three regions, the content of clay minerals exhibits linear correlation with each parameter, implying a close relationship between pore development and clay minerals.

**Table 2:** Relationship between Mineral Composition and Nitrogen Adsorption Parameters in Different Regions^a^

	clay	quartz + feldspar	carbonate
Fengcheng formation	P		
Yanchang formation			
Qingshankou formation	P	N	
Garau formation	P	P	N

a Note: P means linear positive correlation, N means linear negative correlation.

Previous studies have indicated high clay mineral content in both the Qingshankou Formation and Yanchang Formation, with most exceeding 45%.^19,71,72^ However, the types of clay minerals vary.^73^ Interestingly, in this study, the clay content of the Yanchang Formation is slightly higher than that of the Qingshankou Formation. This observation suggests that although clay minerals are crucial for pore development, excessively high clay mineral content may lead to pore development unrelated to clay minerals but more closely associated with other factors.

The porosity characteristics of Chang 7 Member shale within the Ordos Basin exhibit significant dependence on the presence of organic matter and clay minerals. Wang contends that the thermal maturation of organic matter positively influences the early development of petroleum window pores, while clay minerals play a constructive role in enhancing shale porosity. Lacustrine shales undergo three primary types of clay mineral transformations, namely, montmorillite-Illite transformation, montmorillite-Illite-chlorite transformation, and albite-kaolinite-Illite transformation. The transformations of these clay minerals manifest positive impacts on pore volume. However, it is noteworthy that the montmorillite-Illite transformation exhibits some adverse effects on the connectivity of the pore system.^72^

The pores of Qingshankou Formation shale in Songliao Basin are greatly affected by organic matter, thermal maturity, and clay minerals. According to Wang et al., TOC is strongly negatively correlated with pore volume (and porosity) at early maturation and positively correlated at high maturation, indicating that oil generation controls pore formation and significant porosity increases (*R*_o_ ∼ 0.9–1.3%) occur in the oil generation window. The transformation of clay minerals has a great influence on pore formation, and in the transformation process, the smite-Illite transformation has the greatest influence on pore formation in shale. Additionally, an excess of chlorite (>5.0%) is found to positively impact pore formation in clay-rich shale during the transformation process. However, there are differences in clay mineral composition between Chang 7 Member and Qingshankou Formation. Hou et al. analyzed the influence of total clay minerals and Illite content on pore volume and found that Chang 7 Member and Qingshankou shale showed opposite trends, which they believed was caused by the different composition of clay minerals.^73^

Different from the shale rich in organic matter and clay minerals in Chang 7 Member and Qingshankou Formation, the organic matter and clay minerals content in Fengcheng Formation is lower. The pores of Fengcheng Formation in Mahu Sag are mostly solution pores, among which the shale with high content of feldspar + quartz is dominated by solution pores of feldspar, and the shale with high content of carbonate rock is dominated by solution pores of dolomite and calcite.^74^ The sample used in this experiment has a high content of feldspar + quartz, and siliceous shale accounts for the majority of the sample (Figure 3). Therefore, a large number of feldspar pores can be seen under the SEM microscope (Figure 8). The dissolution is an important factor in the formation of shale oil “sweet spot” reservoir in Fengcheng Formation, including the dissolution of terrigenous clastic feldspar particles, feldspar chips in tuffaceous volcanic materials with matrix and carbonate components. Shan et al. believe that there are two main types of dissolution in the study area: one is atmospheric freshwater dissolution, mainly during sedimentary discontinuous or tectonic uplift. The other is organic acid corrosion, which is mainly controlled by organic acid corrosion accompanied by mature hydrocarbon generation of organic matter in source rocks during buried diagenetic period.^74^ Shale with a high content of autogenous felsic minerals, on the one hand, can increase the intergranular pores and specific surface area of rock, and on the other hand, can make shale cement more compact,^75^ which is one of the important factors to increase the brittleness of shale. In the process of felsic mineral enrichment, along with the dissolution and transformation of the original clay matrix and the formation of autogenous silicate minerals, a large number of matrix solution pores and intercrystalline pores are produced. During the diagenetic process, the felsic minerals of Fengcheng Formation can increase both brittleness and porosity of rocks.^70^

## 6 Conclusions

In this study, we combined various methods to study the pore structures of the shale samples from Feng Cheng Formation. Based on this study, we can get the following equations.

(1) The nitrogen adsorption results in the Fengcheng Formation of Mahu Sag indicate a robust correlation between pore parameters and clay minerals, while exhibiting a weaker correlation with quartz + feldspar and carbonate. However, the outcomes from mercury injection tests reveal no significant correlation between mineral composition and porosity, permeability, mean pore radius, and fractal dimension.
(2) The development of organic pores in the Fengcheng Formation of Mahu Sag is minimal, with pore development predominantly observed around feldspar and clay minerals, where the dissolution pores of feldspar are prevalent. Despite the low clay mineral content in the Fengcheng Formation of Mahu Sag, the abundance of feldspar, which can dissolve to generate pores and clay minerals, elucidates the findings from the nitrogen adsorption analysis.
(3) Samples from other wells within the Fengcheng Formation of Mahu Sag exhibit comparable regularities in mineral composition and characteristics to Well X. However, the mineral composition in other geographical areas significantly differs from that observed in Mahu Sag. Typical continental shale reservoirs in China, such as Qingshankou Formation in Songliao Basin and Yanchang Formation in Ordos Basin, generally develop a large amount of clay minerals, which affects the development of pores. However, the Fengcheng Formation in Mahu Sag has little clay mineral content with a large amount of felsic and carbonate minerals. The porosity types and development factors of Fengcheng Formation in Mahu Sag are different from those of other continental shales.

## Data Availability

The data is available throughout the manuscript and Supporting Information.

## Supporting Information Available

Results of XRD mineral quantitative analysis, analysis results of nitrogen adsorption experiment parameters in well X; results of mercury injection test in well X; relationship between different mineral contents and nitrogen adsorption parameters in well A–F, and relationship between different mineral contents and nitrogen adsorption parameters in Yanchang, Qingshankou, and Garau Formation (PDF)
