# Supplementary material for: Detailed Characterization of Pore Results of Continental Shale Reservoir in Fengcheng Formation, Mahu Sag
Source: ACS Omega. 2024 May 14;9(21):22923–40. doi: 10.1021/acsomega.4c02056 (PMC11137716; doi:10.1021/acsomega.4c02056)
Supplement: Supplementary file 1 — ao4c02056_si_001.pdf [file ao4c02056_si_001.pdf]

Detailed characterization of pore results of continental shale reservoir in Fengcheng Formation, Mahu Sag

Zhengchen Zhang[a], Kouqi Liu[a*], Zhenlin Wang[b], Feifei Luo[b], Hong Zhang[a],

[a] Institute of Energy, School of Earth and Space Sciences, Peking University, Beijing, China

[b] Exploration and Development Research Institute of CNPC Xinjiang Oilfield Company, Karamay, China

Email: kouqi.liu@pku.edu.cn

Table S1. Results of XRD mineral quantitative analysis

Sample	Clay	Quartz	Potassium feldspar	Plagioclase	Calcite	Dolomite	Pyrite	Anhydrite	Glauberite
1	7.2	35.8	11.7	22.5	1	16.5	5.3	*	
2	2	10.5	6.3	38.6	20.8	11.9	5.3	4.6	
3	6.1	58.1	2	10.5	19.8		3.5	*	
4	4.8	30.3	1.9	9.2	0.3	51.4	2.1	*	
5	4.6	16.4	4.5	13.8	7.2	50.4	3.1	*	
7	4.6	34.1	5.8	19.8	3.5	27.4	4.8	*	
8	4.5	42.1	3.4	19.6	12.3	13.1	5	*	
9	3.9	55		14.3	18	6	2.8	*	
10	4.1	33.9	3.2	28.9	11.4	9	5.4	4.1	
12	2.6	70.4			0.3	25.8	0.9	*	
13	5.1	17.3	6.5	21.9	2.4	40.8	5.2	0.8	
14	4.3	35	1.5	8.7	2.1	46.1	2.3	*	
15	3.1	29.6	1.5	12.8	35.2	12.4	2.6	2.8	
16	3.5	18.1	4.4	26.4	27	13.7	4.5	2.4	
17	5.1	18.5	4.7	31.1	15.4	11.7	9.1	4.4	
18	4	41.6		16.5	11	20.4	3.7	2.8	
19	4	18.5		16.7	13.4	41.2	2.8	3.4	
20	4.2	18		13.2	0.8	60.4	2.9	0.5	
22	5.4	45.8		18.4	10	15.5	4.1	0.8	
23	3.7	44.5		17.9	21.3	6.1	3.7	2.8	
24	5.9	27.7		34.5	5.3	19.2	6.2	1.2	*
25	5.9	32.4		30.8	7.6	11.2	7.8	4.3	*
26	5.5	31.4	1.5	24.7	13.8	15.5	4.6	3	*
27	3.9	21.4		38.6	1.3	23.2	6.5	5.1	*

28	5.4	22.1	3.9	38.7	2.2	15.1	6.5	4.7	1.4
29	4.1	17.4	3.2	34.7	8.9	24.4	4.7	1.3	1.3
30	6.7	20.1	2.8	26	4.5	26.8	5.8	4.7	2.6
31	6.6	18.4	4.8	43.1	2.7	10.1	6.8	5.4	2.1
32	3.2	16.3		22.6	2.4	50.1	3.6	0.8	1
33	3.4	19.6		26.5	3.3	41.3	3.4	1.1	1.4
34	3.5	22.9		39.2	6.6	16.3	4.9	5.3	1.3
35	4.1	15.3	5.7	39.1	9.9	13.5	6.9	5.5	*
36	4.7	16.8	5.9	37.7	6.7	21.2	5.5	1.5	*
37	5.6	15.5	5.5	35.7	10.5	14.5	7.3	4.5	0.9
38	6	17.9	5.4	36.9	1.3	19.7	6.6	4.9	1.3
39	5.1	14.8	3.9	27.8	9.8	30.9	4.9	1.1	1.7
40	5.6	13.6	4.4	30.7	30	5.1	5.4	3.8	1.4
41	5.3	15.8	4.9	34.1	16.8	12.8	5.8	4.5	*
42	4.9	16.4	6.4	34.5	2.2	27.3	6.8	1.5	*
43	4	10		42	28.3	5.1	6.1	4.5	*
44	4.6	15.9	5.6	29.1	4.5	27.8	5.4	5.2	1.9
45	3.6	12.7	6	31.8	25.8	9.1	5.3	3.5	2.2
46	6	12.8	4.2	32.5	16.3	16	6.7	1.3	4.2
47	4.6	14.5	5.9	26.8	5.2	28.4	6.3	3.6	4.7
48	4.6	12.3	5.2	23.2	10	33.6	4.5	2.7	3.9
49	3.9	13.5	5.3	21	15.2	28.8	4.7	3.7	3.9
50	10	23.3	1.8	19.4	1.8	38.1	3.4	0.9	1.3
51	9.3	26.3	4.4	15.1	4.2	33.3	5	0.8	1.6
52	7.7	17.4	3.5	21.9	21.2	22	4	1.1	1.2
53	10.3	31.4	4.6	16.1	15.2	14	4.5	2.3	1.6
54	3.3	30.1	4.6	18.6	21.4	15.8	4.1	0.8	1.3
55	5.8	44	1	5.7	11.7	29.8	2	*	*
56	4	36.5	1.2	7.7	2.7	46	1.9	*	*
57	3.7	22.8	1.8	30	26.1	7.2	5.2	3.2	*
58	4.6	18.9	1.6	23.3	31.7	11	4.9	3	1
59	6	47.1		15.4	9.9	17.9	3.2	0.5	*
60	4.4	15	4.7	42.1	15.5	7.3	5.6	5.4	*
61	4.1	20	1.7	16.1	37.9	13.9	3.4	2.9	*
62	4.9	16.8	5.1	21.6	35.2	10.5	4.8	0.6	0.5
63	4.4	36	2.6	14	25.8	9.2	3.9	2.2	1.9
64	5.3	22.9	3.4	14.9	1.1	45.3	4.8	0.6	1.7
65	3.3	49	1.8	23.3	5.3	10.9	3.1	3.3	*
66	3.4	25.3	5.4	31.7	17.1	9.1	5	1.2	1.8
67	3.4	36.4		10.2	21.6	22.7	2.5	3.2	*
68	3	15.9	1.7	18	40.7	12.3	5.2	3.2	*
69	4.1	26.7	3.1	18.2	14.9	27.4	4.1	0.6	0.9
70	0.4	1	1.9	55.7	22.7	9.8	2.9	5.6	*
71	1.7	13.3		42.3	19.6	15.6	1.7	5.8	*

72	1.1	35.5	2	22.7	12	20.2	2.3	4.2	*
73	4	22.6	4.4	39.9	10.7	5.2	7.1	4.9	1.2
74	3.1	50.9		21.2	14.6	4.8	4.7	0.7	*
75	2.6	31.1	1.4	10.7	2	49.6	2.2	0.4	*
76	3.8	24.5	1.6	23.6	30.3	8.7	4.8	2.7	*
77	5.7	49.4	1.3	11.2	12.7	16.6	3.1	*	*
78	4.6	31.3	3.2	22	5.1	27.1	4.2	0.8	1.7
79	1.8	18.2	0.6	8.3	63.3	6.3	1.5	*	*
80	6.6	23.4	4.7	10.5	0.5	49.3	3.3	*	1.7
81	4.3	33.2	2.2	13.2	21.2	18.4	3.7	2.9	0.9
82	5.5	34.5	1.2	9.4	35.1	6.9	4	2.2	1.2
83	10	31.8		19.6	8.1	21.8	4.9	0.8	3
84	10.8	33.1	8	22	6	8.7	5	3.5	2.9
85	6.1	23.4	7.4	18	0.6	37.4	4.1	0.6	2.4
86	7.5	23.6	5.4	21.8	6.3	25.2	4.1	3.6	2.5
87	5.1	23.9	5	14.4	28.2	17.7	4	*	1.7
88	5.5	16.6	7	23.3		39.5	5.5	*	2.6
89	13.9	26.4	7.5	14.8		33.2	4.2	*	*
90	3.6	29.1	4	10.4	2.9	45.1	2.9	*	2
91	3.6	29.4	3.9	7.8	1.5	48.5	2.4	*	2.9
92	8	24.3	9.7	17.1	8.7	22.4	5.2	*	4.6
93	3.7	17.5	1.7	12.7	29.8	27.1	2.8	3	1.7
94	5.7	41		16.6		29.4	4.7	*	2.6
95	6.6	41.4	2.1	12	4.1	30.7	3.1	*	*
96	9.4	31.2	13.1	19.6	5.6	13.7	3.3	*	4.1
97	8.2	27.5	7.6	15.5	25.9	7.5	4.8	*	3
98	7.8	15	1.9	11.6	46.4	12.6	3.2	*	1.5
99	10.7	41.4	1.9	9.2	16.8	13.8	4.5	*	1.7
100	8.8	30.8	5.4	9.9	6	33.5	3.7	*	1.9
101	9.6	40.3	6.9	10.6	7.4	18.7	3.9	*	2.6
102	6.2	30.8	4.5	7.9	20.4	25.7	2.7	*	1.8
103	6.7	29.4	2	10.5	19.4	28.3	2.6	*	1.1
104	10.1	37.2	1.4	14.1	21.5	9.3	6.4	*	*
105	19.4	19.5	2.7	21.6	30.4	1.6	4.8	*	*
106	10	27.2	3.2	21.3	25.4	3.5	4.4	3.5	1.5
107	4.1	76.8		1.8	16.2		1.1	*	*
108		42.4	1.5	8	39.2	6.8	2.1	*	*
109	8.8	18.8	3.9	18.5	40.8	5.2	4	*	*
110	4.3	52.4	1.8	10.3	7.4	21.1	2.7	*	*
111	3.4	17	2.8	32.1	24.2	9.2	7	4.3	*
112	4.3	33.5	1.9	17.8	22.6	15	4.2	0.7	*
113	6.5	27.4	1.6	19.7	23.1	15.6	5.5	0.6	*

Table S2. Analysis results of nitrogen adsorption experiment parameters in well X

Sample	Hysteresis loop area	Specific surface area m^2/g	Pore volume mL/g	Mean pore size nm	D1	D2
1	0.62748	5.9601	0.012367	14.2622	2.4652	2.5634
2	0.04388	1.0846	0.002099	24.6974	2.3777	2.4678
3	0.07017	1.594	0.003254	27.3852	2.3748	2.4267
4	0.07666	1.96	0.003854	27.086	2.353	2.438
5	0.19909	2.6828	0.00661	21.8599	2.433	2.4663
6	0.12459	2.0394	0.004864	25.199	2.4145	2.4384
7	0.05628	1.6681	0.00321	28.1237	2.4101	2.4229
8	0.06756	1.6822	0.003497	29.6216	2.3917	2.4158
9	0.18288	1.3646	0.002476	29.9437	2.3972	2.4283
10	0.05455	1.3817	0.002558	30.5423	2.3947	2.4251
11	0.05891	1.3916	0.0029	30.1735	2.4018	2.4101
12	0.12013	1.258	0.002843	27.7226	2.4619	2.4147
13	0.09569	1.4912	0.003343	25.8609	2.4437	2.4339
14	0.11483	1.7318	0.004032	26.1237	2.4306	2.4185
15	0.04721	1.4238	0.002702	29.947	2.3688	2.4318
16	0.03258	1.3333	0.002131	26.4394	2.3597	2.4621
17	0.03373	1.2542	0.002392	29.4821	2.3608	2.4376
18	0.05196	1.617	0.002829	28.5256	2.3747	2.4359
19	0.22585	3.0395	0.006482	17.5865	2.4634	2.5319
20	0.21505	2.606	0.006354	21.2371	2.4593	2.4733
21	0.05458	1.1352	0.002372	28.5123	2.4182	2.4291
22	0.09929	1.9094	0.00399	29.9127	2.3745	2.4158
23	0.05325	1.2475	0.002439	30.9356	2.4012	2.4098
24	0.10407	1.9477	0.004243	30.1773	2.4078	2.4053
25	0.09798	1.8991	0.003936	26.7493	2.4059	2.4402
26	0.06342	1.7189	0.003323	31.1922	2.3824	2.4112
27	0.06657	1.3593	0.002709	29.592	2.4037	2.424
28	0.12695	1.773	0.004041	24.5437	2.4456	2.4476
29	0.12843	1.9648	0.004564	22.2956	2.4314	2.444
30	0.23893	2.4922	0.006192	23.5991	2.4593	2.4361
31	0.14142	1.9436	0.004629	26.1151	2.4525	2.4351
32	0.0635	1.3607	0.002972	25.0132	2.418	2.4266
33	0.18044	2.0988	0.004667	23.1571	2.4632	2.4713
34	0.16029	2.0364	0.004606	22.8425	2.4613	2.456
35	0.13078	2.0938	0.004389	24.1264	2.4394	2.4473
36	0.11353	2.1487	0.004631	24.0499	2.4176	2.4574
37	0.09844	1.9755	0.004081	25.2466	2.3979	2.4473
38	0.11666	2.4275	0.005425	23.9894	2.4069	2.4468
39	0.12256	2.5185	0.005543	23.6323	2.4041	2.4505
40	0.13417	2.609	0.005813	23.437	2.4096	2.4552

41	0.12219	2.6235	0.005959	21.1888	2.4192	2.4563
42	0.10609	2.3653	0.005102	23.3249	2.4414	2.4552
43	0.19173	2.7475	0.006384	21.614	2.4681	2.4684
44	0.20501	3.193	0.007155	21.4267	2.4526	2.4619
45	0.17384	2.8571	0.006313	22.8129	2.4554	2.4656
46	0.13506	2.8355	0.006243	21.0691	2.4472	2.4721
47	0.17114	3.4844	0.007851	20.8045	2.4642	2.4804
48	0.1761	3.169	0.00718	20.415	2.4734	2.4796
49	0.25377	3.588	0.007831	19.9686	2.4828	2.4913
50	0.33967	2.1686	0.005522	25.0873	2.4347	2.4213
51	0.17231	1.5869	0.003068	31.1963	2.4032	2.3949
52	0.19935	1.6759	0.004188	26.6328	2.4075	2.4224
53	1.10111	4.3647	0.009152	13.625	2.4666	2.5775
54	0.0475	0.647	0.001062	35.8245	2.4534	2.3884
55	0.26963	1.3272	0.003176	25.6096	2.4658	2.4358
56	0.17567	1.054	0.002505	28.9068	2.4644	2.3923
57	0.02739	0.8513	0.001245	30.238	2.3972	2.4222
58	0.14712	1.3591	0.002995	30.1457	2.4198	2.4113
59	0.16003	1.1648	0.002672	31.8863	2.425	2.3929
60	0.04209	0.6675	0.001218	27.6659	2.4576	2.4235
61	0.07492	1.2691	0.002926	33.8509	2.4006	2.3766
62	0.12177	1.1582	0.002829	30.1035	2.4346	2.3917
63	0.09089	1.2614	0.002509	31.5554	2.4439	2.3955
64	0.13536	1.3323	0.003002	28.7009	2.4502	2.4034
65	0.03687	1.5777	0.002189	35.0126	2.3856	2.4316
66	0.05386	1.2801	0.002288	27.0358	2.4089	2.4228
67	0.30926	1.5117	0.003706	29.9446	2.4342	2.3974
68	0.07128	1.1071	0.00222	25.619	2.3986	2.4383
69	0.17052	1.1898	0.002667	20.9563	2.4305	2.4635
70	0.01212	0.2738	0.000581	33.498	2.3903	2.4245
71	0.04153	0.568	0.001128	28.328	2.4223	2.4274
72	0.04941	0.9066	0.00167	29.135	2.3852	2.4245
73	0.11464	1.2146	0.003147	26.7871	2.3902	2.3997
74	0.38271	2.422	0.006564	35.4737	2.385	2.3555
75	0.26237	1.6517	0.004231	26.1968	2.4226	2.4062
76	0.22651	1.7418	0.003973	31.2147	2.4109	2.3848
77	0.56173	2.6541	0.007049	22.4688	2.4401	2.4426
78	0.1737	1.2022	0.002968	24.2857	2.44	2.4443
79	0.12605	0.8715	0.001942	26.21	2.4497	2.4255
80	0.45869	2.1484	0.005319	24.1284	2.4473	2.4421
81	0.60009	1.7778	0.004589	23.5629	2.463	2.4625
82	0.54646	2.1063	0.005312	22.9828	2.451	2.4648
83	0.83879	5.0311	0.009402	12.5319	2.4856	2.6118
84	0.80423	4.4351	0.008627	13.1323	2.4789	2.5943

85	0.43921	2.0229	0.005026	19.8893	2.4595	2.4951
86	0.66912	5.0918	0.008514	11.2758	2.5131	2.6496
87	0.23239	1.7132	0.004032	23.2284	2.4243	2.4465
88	0.16174	1.1817	0.00278	24.5948	2.4398	2.4371
89	0.91202	4.4415	0.009372	14.9511	2.4411	2.5512
90	0.09513	1.7385	0.003426	25.0183	2.3418	2.4475
91	0.10827	2.1731	0.004217	18.4503	2.3645	2.5155
92	0.72256	5.1889	0.010253	13.1645	2.4555	2.5868
93	0.29699	1.5889	0.003868	23.0915	2.429	2.4476
94	0.29359	1.7051	0.004259	22.9777	2.4294	2.4465
95	0.36961	1.5842	0.00417	25.0147	2.4578	2.4392
96	0.61599	2.1671	0.006087	23.9926	2.4692	2.4302
97	0.23268	1.5742	0.004122	26.2991	2.4275	2.426
98	0.52087	2.5657	0.006794	19.144	2.4613	2.4885
99	0.48026	2.3216	0.006177	23.8101	2.4676	2.453
100	0.6795	4.4899	0.009673	14.8979	2.4325	2.5714
101	0.72997	5.0868	0.010503	13.0466	2.4302	2.5833
102	0.46476	2.6103	0.006877	21.4029	2.459	2.4673
103	0.51776	2.687	0.006905	18.809	2.445	2.4999
104	0.34526	1.8684	0.005035	27.3315	2.4575	2.4107
105	0.81832	7.9956	0.015261	15.375	2.4518	2.5506
106	0.49888	2.8717	0.007674	27.5454	2.4478	2.4085
107	0.08708	1.1276	0.002193	34.7506	2.3799	2.3839
108	0.08965	0.6817	0.001634	27.614	2.4686	2.4091
109	0.47739	3.6626	0.009415	21.6456	2.4523	2.466
110	0.02666	0.9089	0.001572	27.0721	2.3775	2.4607
111	0.03208	0.7967	0.001378	29.6756	2.447	2.426
112	0.03037	1.4645	0.00207	35.8561	2.3766	2.4176
113	0.063	1.1584	0.002392	34.7928	2.4111	2.3914

Table S3. Results of mercury injection test in well X

Sample	Permeability mD	Porosity %	Mean pore radius μm	Fractal dimension
2	0.000798	2.38	0.008756	2.4158
3	0.000312	3	0.007787	2.5099
4	0.000241	3.32	0.009154	2.5032
7	0.028479	4.43	0.022364	2.51
10	0.000577	4.19	0.012084	2.4763
14	0.057738	2.39	0.009785	2.3896
19	0.000226	2.45	0.029237	2.6549
25	0.000149	2.19	0.007561	2.4954
26	0.000146	2.58	0.007683	2.4865
33	0.000214	1.11	0.006388	2.3667

34	0.000238	2.16	0.011448	2.5162
35	0.000962	1.7	0.006473	2.3725
36	0.000412	3.56	0.017299	2.4604
37	0.000261	1.15	0.00854	2.5486
42	0.000521	1.42	0.008303	2.4296
43	0.000242	0.19	0.0082	2.4829
50	0.0279	0.79	0.009625	2.4628
51	0.000424	3.491	0.012341	2.5465
52	0.00642	2.134	0.010621	2.4591
53	0.00916	0.181	0.010285	2.5751
54	0.0011	5.56	0.01293	2.5766
56	0.0008	1.34	0.008086	2.5199
57	0.001	5.61	0.011467	2.5009
58	0.00143	1.338	0.010536	2.4868
62	0.0009	3.19	0.009054	2.5349
63	0.0768	2.181	0.009848	2.5387
64	0.0386	4.57	0.008986	2.4311
67	0.000772	1.626	0.007153	2.4622
68	0.000396	0.747	0.006161	2.3773
70	0.0673	0.546	0.023168	2.5799
77	0.069	3.07	0.008815	2.468
78	0.0059	1.01	0.007555	2.4141
84	0.000562	2.219	0.008331	2.471
85	0.000341	1.628	0.008859	2.4223
87	0.00126	0.897	0.00824	2.5417
88	0.000751	2.188	0.006488	2.4293
89	0.000702	0.567	0.005864	2.3494
90	0.0005	5.46	0.011203	2.4769
91	0.0033	2.19	0.009346	2.4555
92	0.104	1.695	0.007374	2.4488
93	0.000535	1.739	0.006866	2.5302
96	0.0616	1.58	0.006892	2.402
97	0.1266	3.31	0.007744	2.3822
98	0.0013	4.42	0.006748	2.3844
101	0.0023	1.19	0.007024	2.4353
102	0.00207	2.992	0.006336	2.5272
104	0.0009	3.68	0.012685	2.6241
106	0.103	4.511	0.009341	2.524
109	0.000398	2.087	0.008852	2.4627

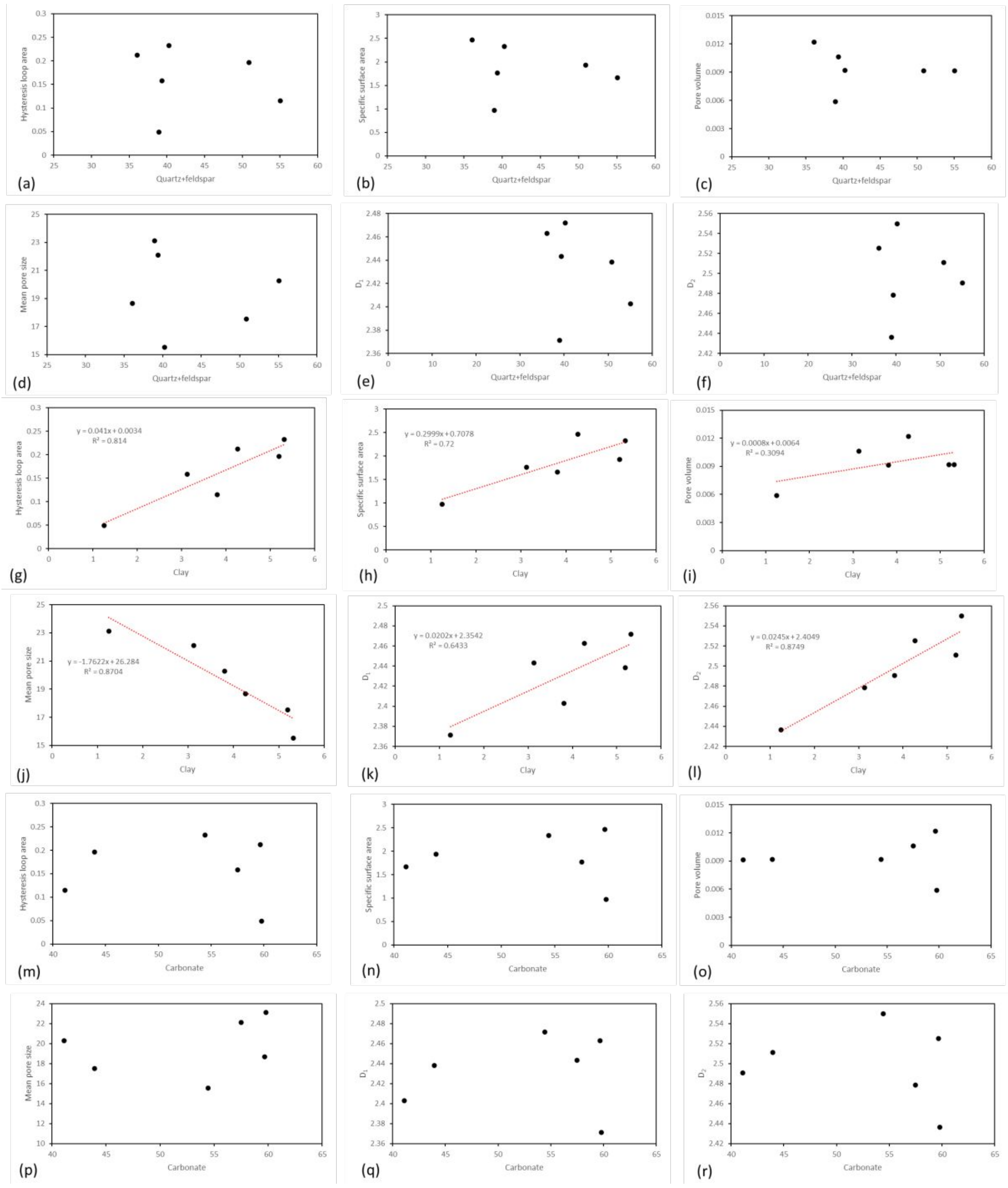

Figure S1. Relationship between different mineral contents and nitrogen adsorption parameters in well A. (a-f) the relationship between quartz + feldspar content and hysteresis loop area, specific surface area, pore volume, mean pore size, D_1 and D_2; (g-l) The relationship between clay mineral content and hysteresis loop area, specific surface area, pore volume, mean pore size, D_1 and D_2. (m-r) Relationship between carbonate content and hysteresis loop area, specific surface area, pore volume, mean pore size, D_1 and D_2

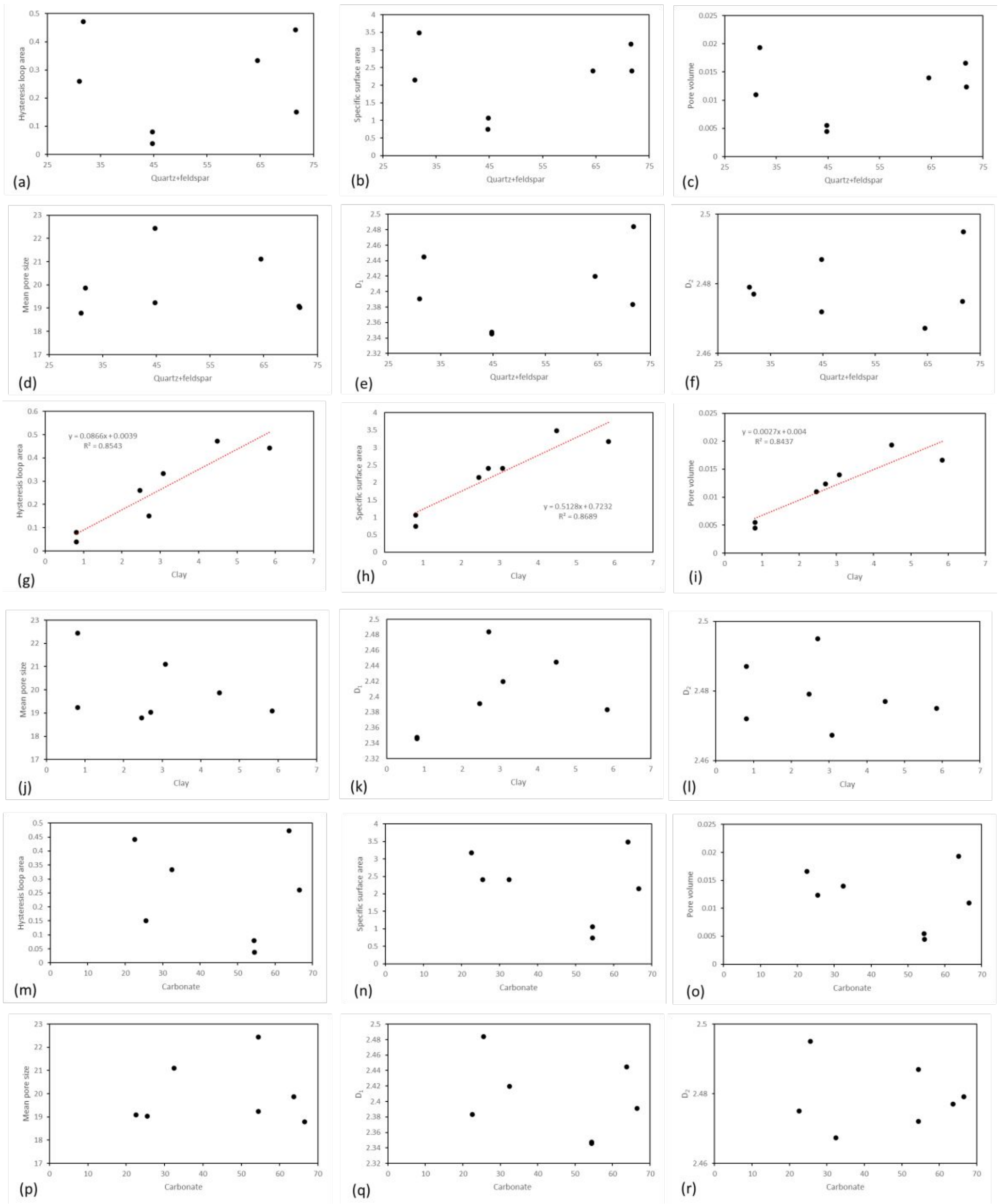

Figure S2. Relationship between different mineral contents and nitrogen adsorption parameters in well B. (a-f) the relationship between quartz + feldspar content and hysteresis loop area, specific surface area, pore volume, mean pore size, D_1 and D_2; (g-l) The relationship between clay mineral content and hysteresis loop area, specific surface area, pore volume, mean pore size, D_1 and D_2. (m-r) Relationship between carbonate content and hysteresis loop area, specific surface area, pore volume, mean pore size, D_1 and D_2

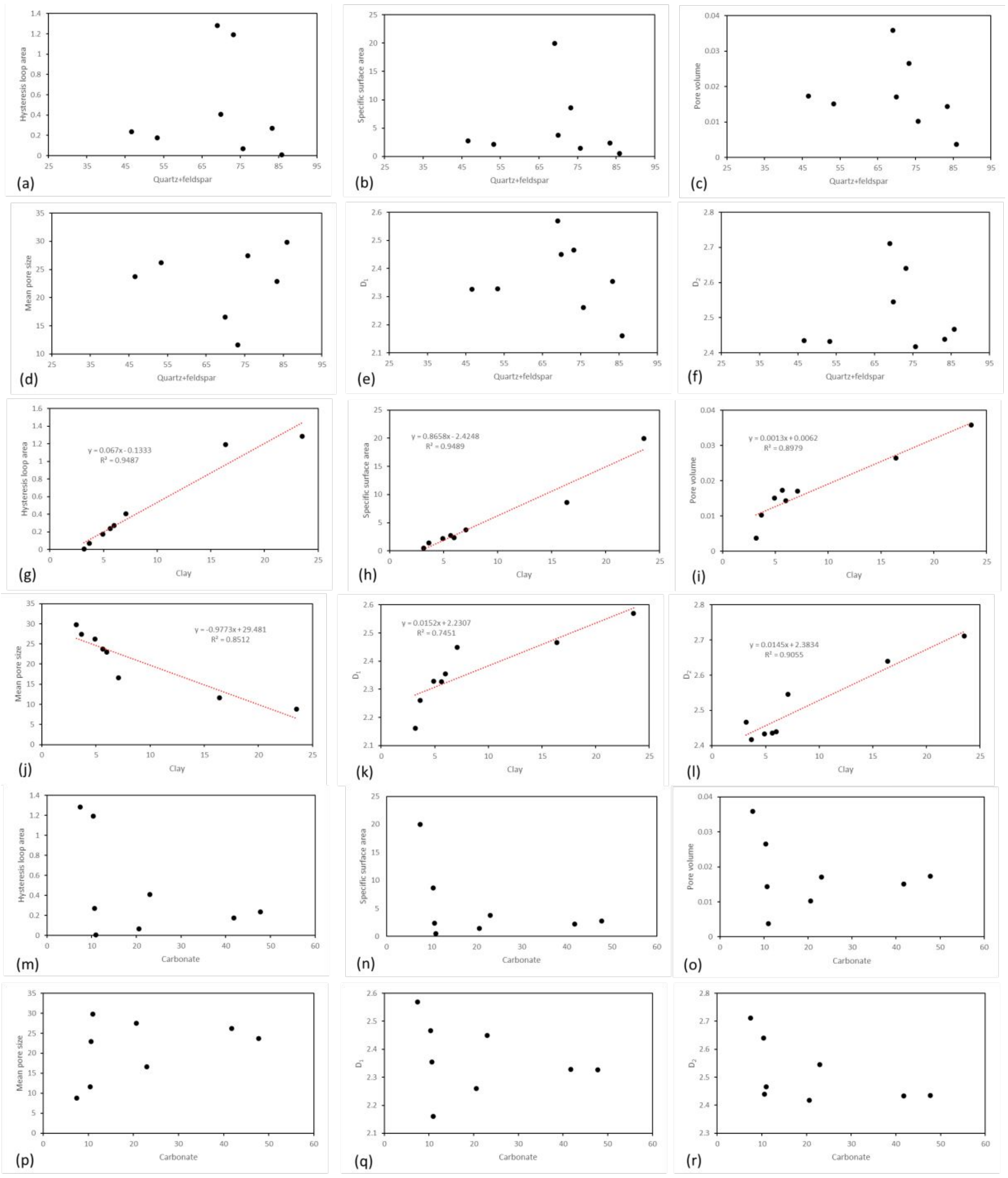

Figure S3. Relationship between different mineral contents and nitrogen adsorption parameters in well C. (a-f) the relationship between quartz + feldspar content and hysteresis loop area, specific surface area, pore volume, mean pore size, D_1 and D_2; (g-l) The relationship between clay mineral content and hysteresis loop area, specific surface area, pore volume, mean pore size, D_1 and D_2. (m-r) Relationship between carbonate content and hysteresis loop area, specific surface area, pore volume, mean pore size, D_1 and D_2

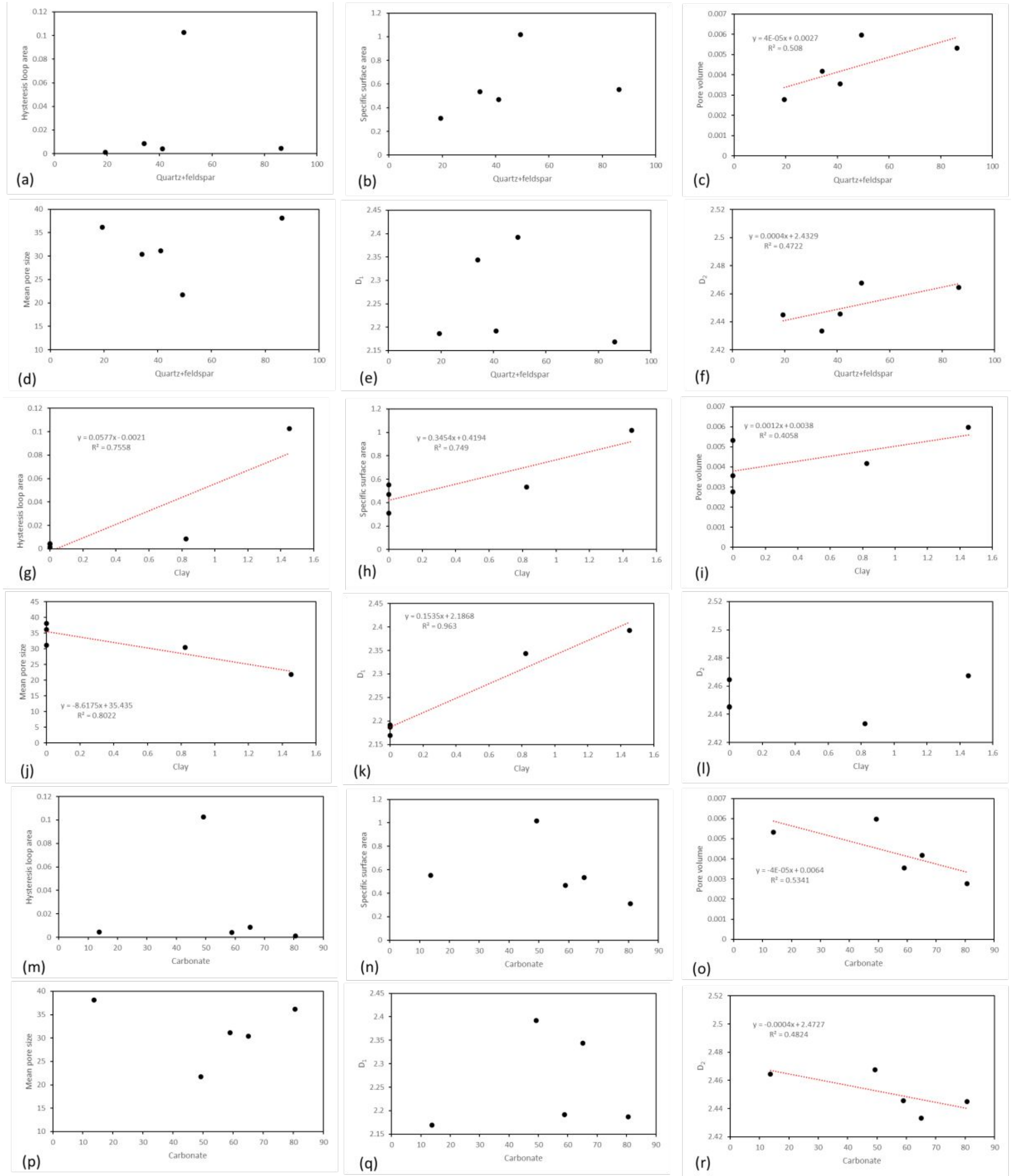

Figure S4. Relationship between different mineral contents and nitrogen adsorption parameters in well D. (a-f) the relationship between quartz + feldspar content and hysteresis loop area, specific surface area, pore volume, mean pore size, D_1 and D_2; (g-l) The relationship between clay mineral content and hysteresis loop area, specific surface area, pore volume, mean pore size, D_1 and D_2. (m-r) Relationship between carbonate content and hysteresis loop area, specific surface area, pore volume, mean pore size, D_1 and D_2

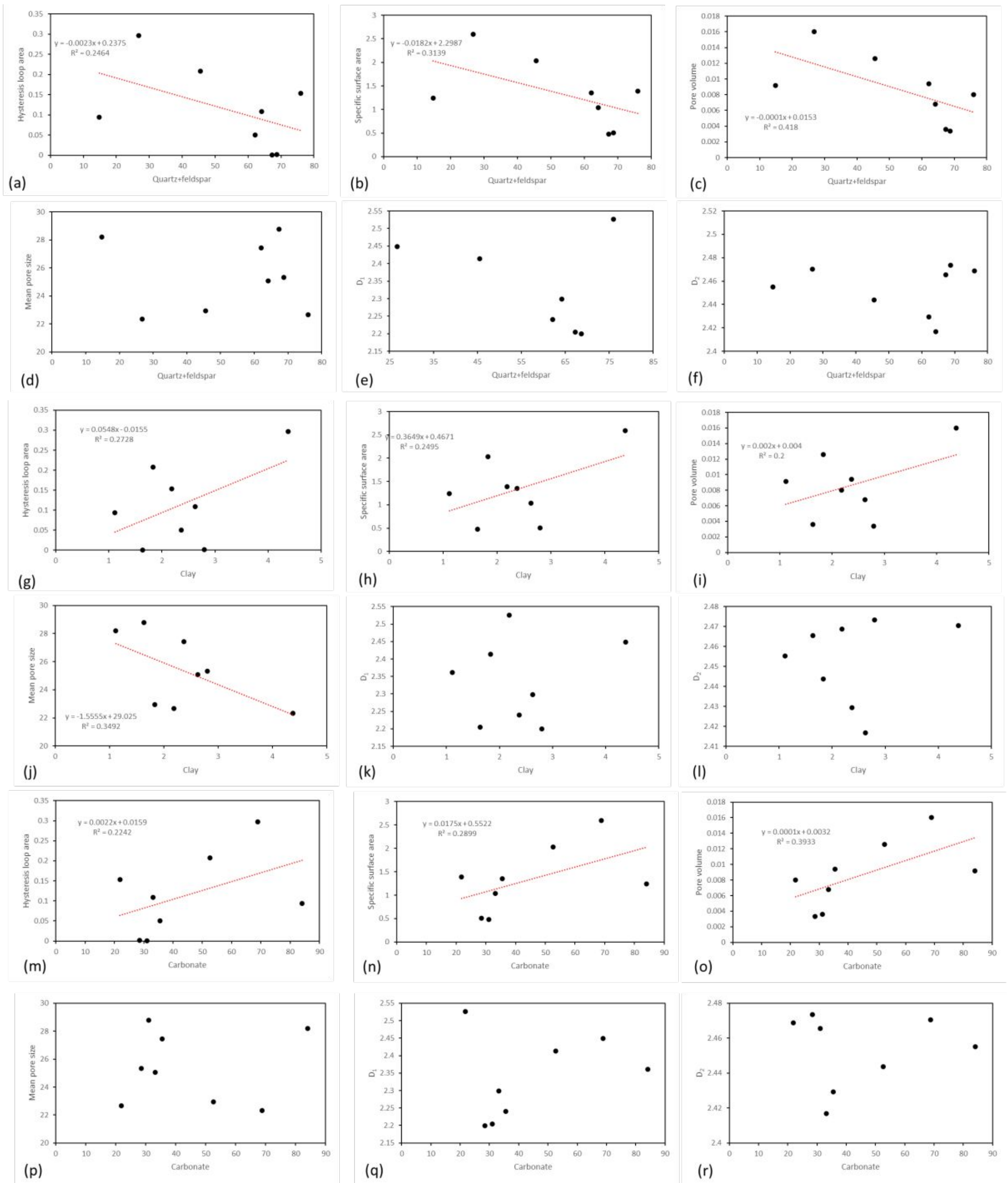

Figure S5. Relationship between different mineral contents and nitrogen adsorption parameters in well E. (a-f) the relationship between quartz + feldspar content and hysteresis loop area, specific surface area, pore volume, mean pore size, D_1 and D_2; (g-l) The relationship between clay mineral content and hysteresis loop area, specific surface area, pore volume, mean pore size, D_1 and D_2. (m-r) Relationship between carbonate content and hysteresis loop area, specific surface area, pore volume, mean pore size, D_1 and D_2

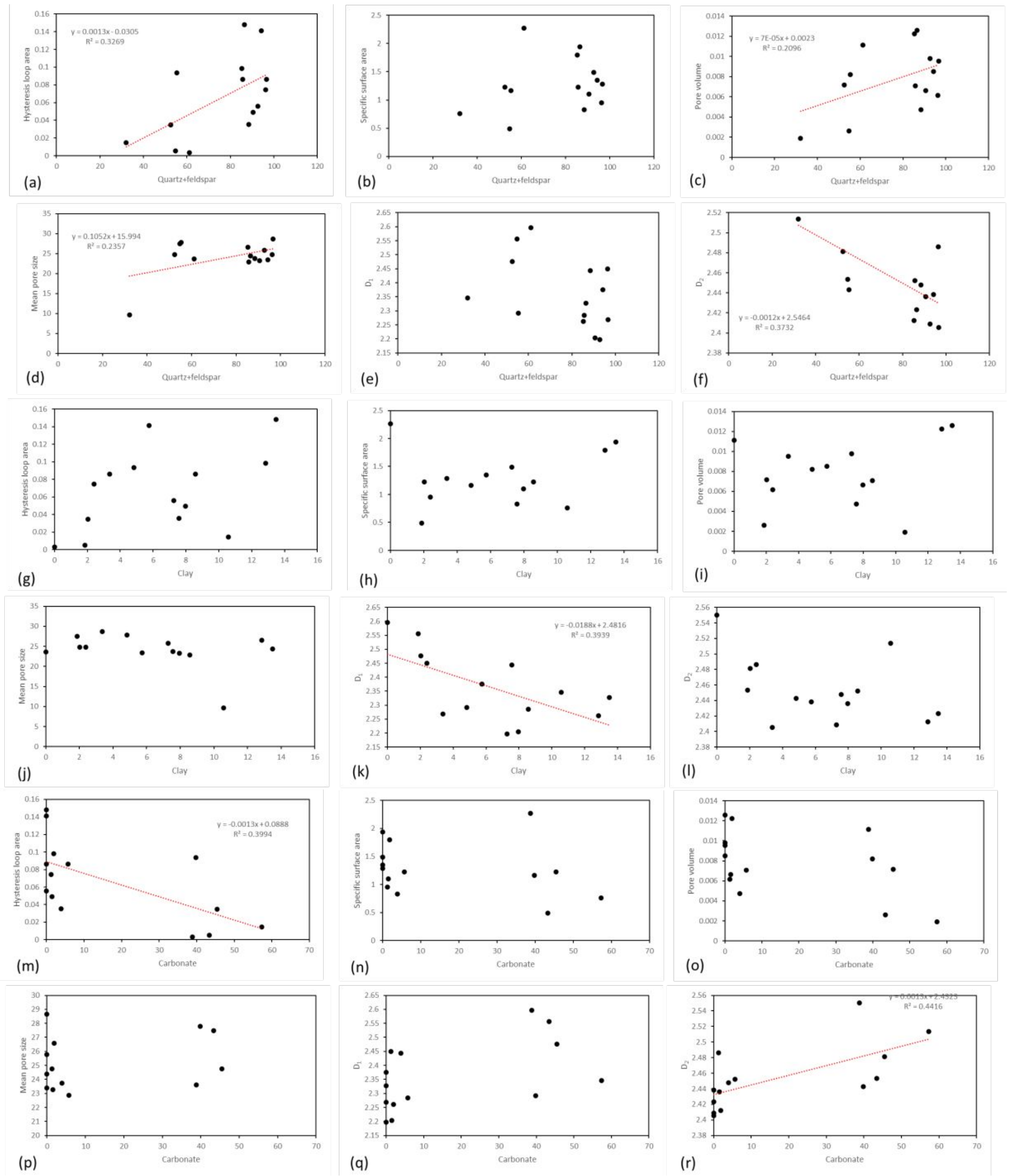

Figure S6. Relationship between different mineral contents and nitrogen adsorption parameters in well F. (a-f) the relationship between quartz + feldspar content and hysteresis loop area, specific surface area, pore volume, mean pore size, D_1 and D_2; (g-l) The relationship between clay mineral content and hysteresis loop area, specific surface area, pore volume, mean pore size, D_1 and D_2. (m-r) Relationship between carbonate content and hysteresis loop area, specific surface area, pore volume, mean pore size, D_1 and D_2

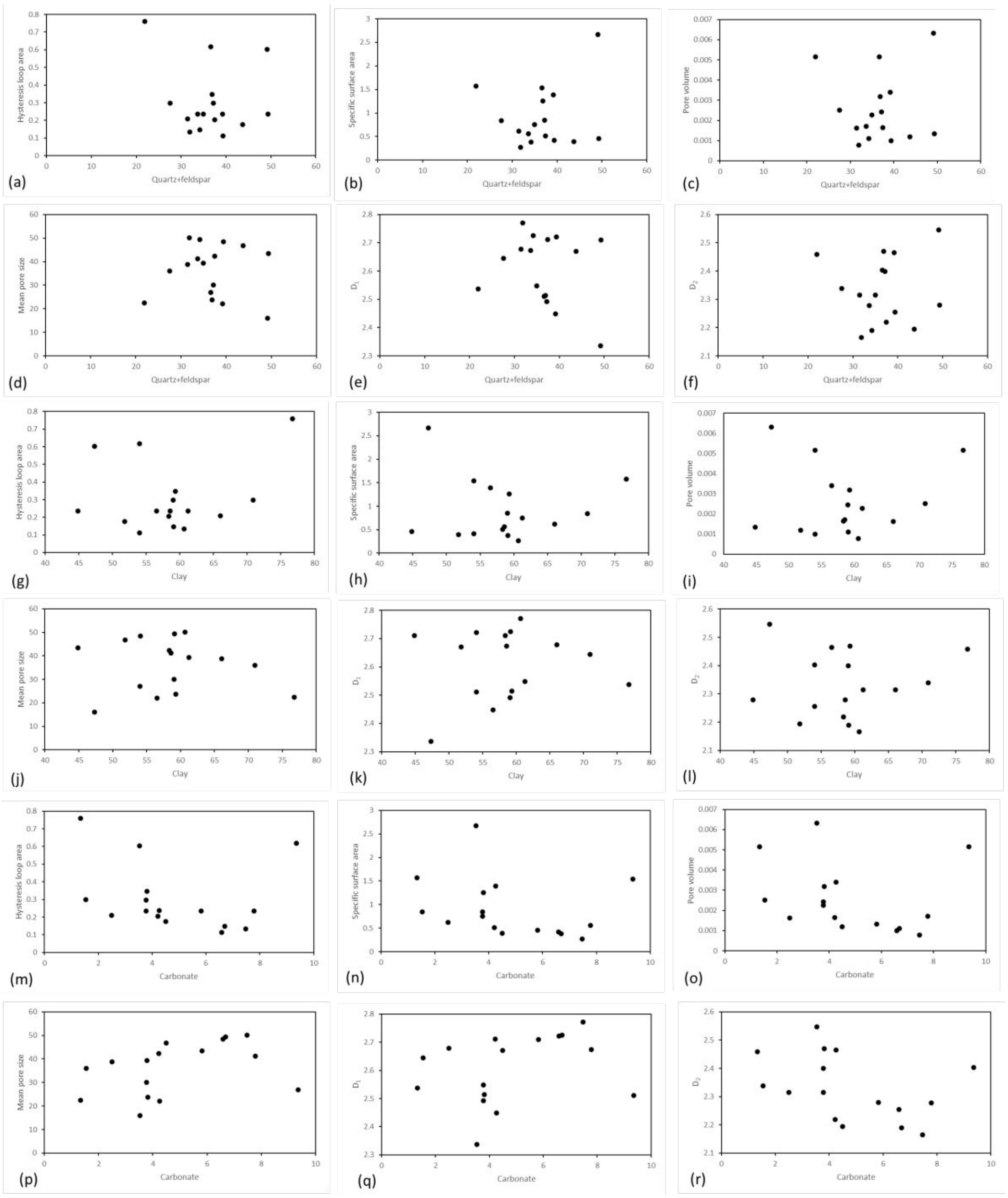

Figure S7. Relationship between different mineral contents and nitrogen adsorption parameters in Yanchang Formation. (a-f) the relationship between quartz + feldspar content and hysteresis loop area, specific surface area, pore volume, mean pore size, D_1 and D_2; (g-l) The relationship between clay mineral content and hysteresis loop area, specific surface area, pore volume, mean pore size, D_1 and D_2. (m-r) Relationship between carbonate content and hysteresis loop area, specific surface area, pore volume, mean pore size, D_1 and D_2

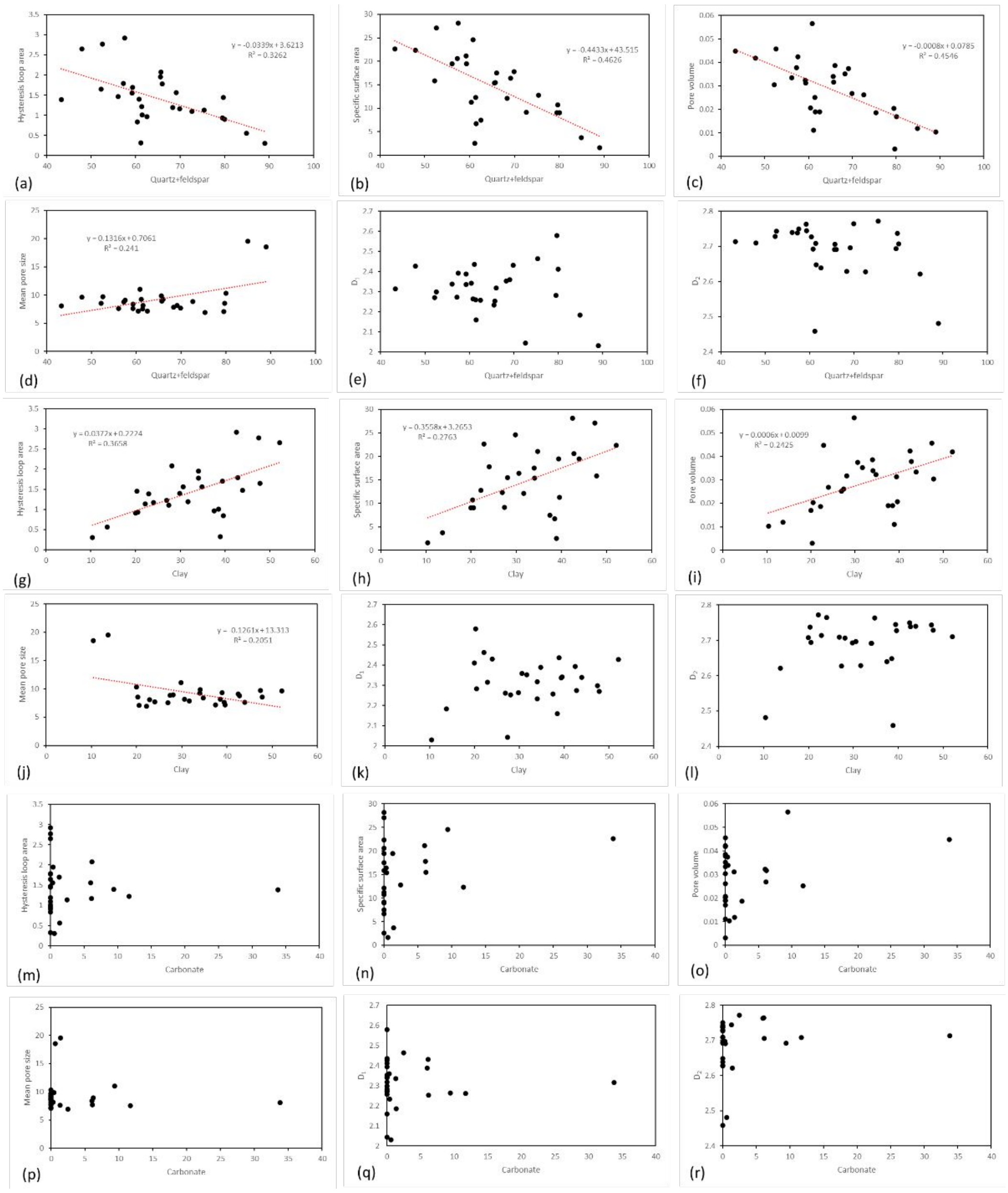

Figure S8. Relationship between different mineral contents and nitrogen adsorption parameters in Qingshankou Formation. (a-f) the relationship between quartz + feldspar content and hysteresis loop area, specific surface area, pore volume, mean pore size, D_1 and D_2; (g-l) The relationship between clay mineral content and hysteresis loop area, specific surface area, pore volume, mean pore size, D_1 and D_2. (m-r) Relationship between carbonate content and hysteresis loop area, specific surface area, pore volume, mean pore size, D_1 and D_2

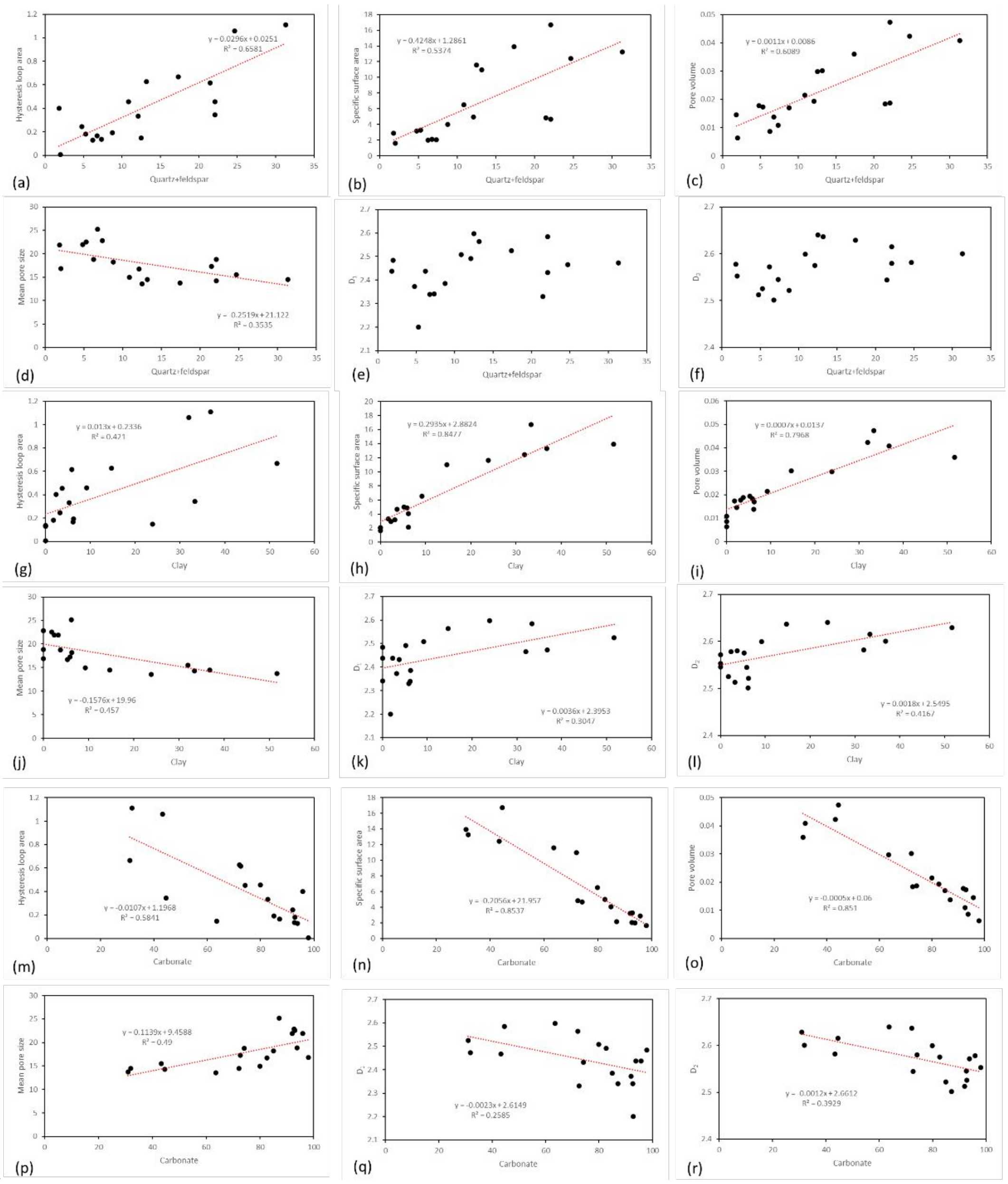

Figure S9. Relationship between different mineral contents and nitrogen adsorption parameters in Garau Formation. (a-f) the relationship between quartz + feldspar content and hysteresis loop area, specific surface area, pore volume, mean pore size, D_1 and D_2; (g-l) The relationship between clay mineral content and hysteresis loop area, specific surface area, pore volume, mean pore size, D_1 and D_2. (m-r) Relationship between carbonate content and hysteresis loop area, specific surface area, pore volume, mean pore size, D_1 and D_2
